# Supplementary material for: Association of initiating CYP2D6-metabolized opioids with risks of adverse outcomes in older adults receiving antidepressants: A retrospective cohort study
Source: PLoS Med. 2025 Jun 2;22(6):e1004620. doi: 10.1371/journal.pmed.1004620 (PMC12129234; doi:10.1371/journal.pmed.1004620)
Supplement: S1 Text — (DOCX) [file pmed.1004620.s002.docx]

**Study Aims:** To determine associations between concurrent use of opioids with antidepressants (ADs) and clinical and safety outcomes in older nursing home (NH) patients.

- Clinical outcomes: pain, physical function, depression.
- Opioid-related adverse event (ORAE) outcomes: pain-related hospitalizations, pain-related emergency department visits, diagnosis of opioid use disorder, and diagnosis of opioid overdose.

**Research design:** Retrospective cohort study

**Data source:** 100% Medicare-MDS 3.0 from 2010-2021 for the NH population

**Sample selection:**

1. Concurrent use of antidepressants and CYP2D6-metabolized opioids during 1/1/2011-12/31/2020
   1. CYP2D6-metabolized opioids included codeine, hydrocodone, oxycodone, and tramadol
   2. The cohort entry date was defined as day 1 of initiating antidepressants or opioids, with no such prescription fills dispensed within 6 months preceding the cohort entry date.
   3. Restricting to treatment episodes of concomitant use to those in which opioids were initiated on existing antidepressants (i.e., opioid-trigged interactions)
2. Aged ≥65 years on the cohort entry date
3. Stayed at least 100 days in NHs before the cohort entry date (to capture long-term NH residents)
4. Had at least one of these three chronic pain conditions: a) musculoskeletal pain; b) neuropathic pain; or c) idiopathic pain diagnosed within 1 month before the cohort entry date
5. Had at least one of the conditions that are FDA-approved or off-label use for antidepressants within 6 months before the cohort entry date (baseline).
6. Had use of antidepressants for at least 30 days during the 6-month baseline (to capture existing antidepressant users)
7. Had survived during the 6-month baseline period
8. Had continuously enrolled in Parts A/B/PDP and had no HMO/EMP coverage during the 6-month baseline period
9. Had no hospital or skilled nursing facility stay during the 6-month baseline period (because no prescription data are available)
10. Had ≥1 MDS assessment during the 6-month baseline period
11. Had continuous NH stay throughout the 6-month baseline period
12. Had no cancer, hospice care, and palliative care during 6 months during the 6-month baseline period.
13. Had no coma or severe cognitive impairment (defined as having a Cognitive Performance Scale score of 5 or 6 from MDS 3.0) during the 6-month baseline period.

*Follow-up*: From cohort entry until death, Medicare disenrollment, NH discharge, or study end (12/31/2021)

Final samples: Present sample selection in a flow chart (i.e., Figure 1). The final sample after applying the 12 inclusion criteria will be used for analysis of ORAE outcomes. From this sample, we further required to have ≥1 MDS assessment during follow-up for analysis of clinical outcomes that are measured from the MDS.

**Clinical Outcomes**

Three clinical outcomes –pain, physical function, and depressive symptoms—will be measured using MDS 3.0 outcomes***.***

- *MDS 3.0 pain* is measured by residents’ recall of the worst pain in the last 5 days using a numeric rating scale score (NRS) of 0 to 10 (no to worst pain) or a categorical verbal descriptor scale (VDS) with 4 response choices (no, mild, moderate, or severe pain).^1^ The two pain scale scores will be combined based on an empirically validated crosswalk^2^ and classified into 4 categories: no (0), mild (1-4), moderate (5-7), and severe (8-10) pain.^3^ Pain intensity of non-verbal residents is assessed by nursing staff who use the Checklist of Nonverbal Pain Indicator (CNPI). For comparison purposes, the CNPI score will be classified as no (none of the behaviors), mild (1 behavior), moderate (2 behaviors), and severe (3 or 4 behaviors) to indicate pain severity.
- *MDS 3.0 physical function* is measured by the 5-item (i.e., dressing, bathing, eating, toileting, and transferring) self-care activities of daily living (ADLs), with each item scored from 0 (total independence) to 4 (total dependence). The total ADL score ranges from 0 to 20, with higher scores indicating worse physical function.
- *MDS 3.0 depression status* is measured by the PHQ-9, with each of the 9 items scored from 0 (not at all) to 3 (nearly every day). The total score ranges from 0 to 27, with higher scores indicating worse depression.

**ORAE outcomes**

Four ORAE outcomes—pain-related hospitalizations, pain-related emergency department (ED) visits, OUD, and OD—will be measured using Medicare claims data. Hospitalizations and ED visits due to chronic pain and any cause will be determined using Medicare inpatient data for each beneficiary. Chronic pain–related hospitalizations will be defined as inpatient encounters with primary or secondary diagnostic codes for chronic pain. *OUD and OD* will be captured from Medicare inpatient or outpatient encounters coded with opioid dependence, abuse, or OD. *Opioid-related and all-cause deaths* are captured via National Death Index data. We will convert *ICD-9* to *ICD-10* codes.

**Concomitant use of ADs and opioids** (*key exposure*): Exposure to concurrent use of opioids and ADs will be captured on each patient-day of follow-up based on fill date and days’ supply of prescription drugs from Medicare Prescription Part D files. We will define concurrent use as an overlap of an opioid and a D of interest for at least 1 day. For each concurrent-use episode, we will: (1) classify ADs into CYP2D6-inhibiting and CYP2D6-neutral agents (i.e., reference) according to sources of databases, including FDA, Micromedex, Lexicomp, and Drug Interaction Database (DIDB®). The following table lists the opioids and AD drugs of interest and classification. Patients who received concomitant use of CYP2D6-metabolized opioids and CYP2D6-inhibiting ADs are classified as the study group, whereas patients who received concomitant use of CYP2D6-metabolized opioids and CYP2D6-neutral ADs are classified as the comparison group.

**Table 1: List of opioids and AD drugs**

| **Opioids** | **AD drugs** |
| --- | --- |
| *CYP-metabolized opioids*: Oxycodone, tramadol, hydrocodone, fentanyl, methadone, codeine | *CYP2D6 inhibitors (strong/moderate)^:^*  SNRIs: duloxetine,  SSRIs: fluoxetine, paroxetine  TCAs: amitriptyline, amoxapine, clomipramine, desipramine, doxepin, imipramine, nortriptyline, trimipramine  Others: mirtazapine, bupropion, vortioxetine |
|  | *CYP2D6-neutrals (Weak/no):*  SNRIs*: desvenlafaxine, levomilnacipran, milnacipran, venlafaxine  SSRIs: sertraline, citalopram, citalopram, fluvoxamine  TCAs*: maprotiline, protriptyline  Others: trazodone, vilazodone, nefazodone, |

**Covariates measured during the 6-month baseline period are detailed as follows:**

1. *Demographics*: age (65-74, 75-84, 85+), sex, race/ethnicity (Whites, Blacks, and Others), low-income subsidy status (Yes vs no), place of residence (based on five-digit ZIP codes and classified as South, Northeast, Midwest, and West), all of which are derived from Medicare Beneficiary Summary File.
2. *Health status*: Tobacco or alcohol use disorder (Yes vs no, assessed based on the diagnostic algorithm for both conditions developed by the Chronic Condition Warehouse, <https://www2.ccwdata.org/web/guest/condition-categories>) and drug use disorder.
3. *Chronic pain diagnosis*: classified as musculoskeletal pain (Yes vs no), neuropathic pain (Yes vs no), or idiopathic pain (Yes vs no). See ICD-9 or ICD-10 codes in the supplemental file. Note that an individual can have more than one type of chronic pain conditions.
4. *Clinical conditions* that may affect opioid treatment, include mental health disorders, diabetes, cardiovascular diseases, hypertension, pulmonary conditions, kidney disease, gastrointestinal tract disorder, liver disease, epilepsy, neurodegenerative conditions.
5. *Health care utilization*: any hospital stay (yes vs no) and any emergency department visit (yes vs no),
6. *Pain* management: use of drug or non-drug intervention (from MDS 3.0), use of PRN pain medications (from MDS 3.0), receipt of procedures and therapies for chronic pain management, use of prescription nonopioids, and use of adjuvant analgesics.
7. *Medication*-*related factors*: polypharmacy (defined as the use of > 4 distinct generic drugs simultaneously), use of central nervous system medications (including benzodiazepine, non-benzodiazepine, anticonvulsants, antidepressants, antipsychotics, anxiolytics), duration of AD use, use of other CYP2D6 inhibitors, use of other CYP2D6 inducers, and use of other CYP3A4 inhibitors.
8. NH-facility factors derived from LTCFocus (https://ltcfocus.org/): total number of beds, profit status, chain membership, having special care unit, and geography (metropolitan, micropolitan, or rural, determined based on rural-urban continuum codes).
9. Other variables: baseline clinical outcomes, cognitive function, duration of antidepressant use, and year of cohort entry.

**Pre-planned data analyses:** Our overall analytic approach includes descriptive analyses, inverse probability of treatment weighting (IPTW) to balance covariates between the study and comparison group, outcome models for the clinical and ORAE outcomes, and sensitivity analyses. All tests are two-sided with a statistical significance of P < .05. Our analytical plan is detailed as follows:

1. Descriptive analysis:

Table 1.1: Describe baseline characteristics of clinical and demographic characteristics of the Sample before and after the IPTW for the analysis of ORAE outcomes, overall and by the study vs comparison group.

Table 1.2: Describe baseline characteristics of clinical and demographic characteristics of the Sample before and after the IPTW for the analysis of clinical outcomes, overall and by the study vs comparison group.

1. Final models:

Table 2: Associations of Residents Receiving Concomitant Use of CYP2D6-Metabolized Opioids with Existing CYP2D6-Inhibiting Antidepressants (Study Group) vs. CYP2D6-Neutral Antidepressants (Comparison Group) With Clinical Worsening and Opioid-Related Adverse Outcomes.

Table 3: Unadjusted and Adjusted Associations of Concomitant Use of Antidepressants and CYP2D6-Metabolized Opioids With Clinical Worsening and Opioid-Related Adverse Outcomes, Stratified By Antidepressant Therapeutic Classes With vs. Without Additional Analgesic Effects

1. Supplemental tables:

S1 Table. Medications of Interest Considered in the Study

S2 Table. *ICD-9-CM, ICD-10-CM,* or Procedure Codes for Disease Conditions and Service Care Considered in the Study

S3 Table. Study Covariates, Definitions, and Measurement Sources and Windows

S4 Table. Clinical and Demographic Characteristics of Eligible Patients With at Least 1 MDS 3.0 in Follow-up and Received CYP2D6-Opioids Concomitantly with CYP2D6-Inhibiting vs. CYP2D6-Neutral Antidepressants

S5 Table. Quarterly Associations of Concomitant Use of CYP2D6-Metabolized Opioids and Antidepressants with Clinical Worsening Outcomes From Baseline to Follow-up

S6 Table. Unadjusted and Adjusted Associations of Concomitant Use of CYP2D6-Metabolized Opioids and Antidepressants with Clinical Worsening and Opioid-Related Adverse Outcomes, Stratified By Specific CYP2D6-metabolized Opioids

S7 Table. Associations of Concomitant Use of CYP2D6-Metabolized Opioids and Antidepressants with Clinical Worsening and Opioid-Related Adverse Outcomes, Adjusting for Censoring Due to Death via Inverse Probability of Censoring Weighting.

S8 Table. Sensitivity Analysis of Including Eligible Residents without Use of Other CYP2D6 Medications at Baseline

References:

1. Saliba D, Buchanan J. Making the investment count: revision of the Minimum Data Set for nursing homes, MDS 3.0. *J Am Med Dir Assoc*. Sep 2012;13(7):602-10. doi:10.1016/j.jamda.2012.06.002

2. Edelen MO, Saliba D. Correspondence of verbal descriptor and numeric rating scales for pain intensity: an item response theory calibration. *J Gerontol A Biol Sci Med Sci*. Jul 2010;65(7):778-85. doi:10.1093/gerona/glp215 glp215 [pii]

3. Moore RA, Straube S, Aldington D. Pain measures and cut-offs - 'no worse than mild pain' as a simple, universal outcome. *Anaesthesia*. Apr 2013;68(4):400-12. doi:10.1111/anae.12148

**SAS Codes for Final Analytical Analysis**

/*Clinical outcomes

1. diff_pain_d: worsening pain (1: yes vs 0: no)

2. diff_adl_d: worsening physical function (1: yes vs 0: no)

3. diff_phq9_d: worsening depression (1: yes vs 0: no)

Key exposure: case: (1: study group, 0: comparison group)

Other variables:

--quarter: 4 quarters (1, 2, 3, 4)

--IPTW weight

--bene_id_n: encrypted ID number represents each episode for each resident.

--final_cl: final analytical dataset for clinical outcomes (at the resident-quarter level)

*/

%macro out (out);

/*crude*/

PROC GENMOD data=final_cl;

CLASS bene_id_n &out (ref='0') case (ref='0') quarter (ref='1');

MODEL &out=case quarter/dist=binomial link=log;

REPEATED subject=bene_id_n /type=unstr;

ODS OUTPUT GEEEmpPEst=_crude;

run;

data _crude_1;

retain PARM Level1 RR RR_L RR_U Probz;

set _crude;

RR=round (exp (estimate), 0.01);

RR_L=round (exp (LOWERCL), 0.01);

RR_U=round (exp (UPPERCL), 0.01);

keep RR: PARM Probz LEVEL1;

run;

/*weighted*/

PROC GENMOD data=final_cl;

CLASS bene_id_n &out (ref='0') case (ref='0') quarter (ref='1');

MODEL &out=case quarter/dist=binomial link=log ;

REPEATED subject=bene_id_n /type=unstr;

WEIGHT IPTW;

ODS OUTPUT GEEEmpPEst=_adj;

data _adj_1;

set _adj;

retain PARM Level1 RR_a RR_L_a RR_U_a Probz;

RR_a=round (exp (estimate), 0.01);

RR_L_a=round (exp (LOWERCL), 0.01);

RR_U_a=round (exp (UPPERCL), 0.01);

rename Probz=Probz_a;

keep RR: PARM Probz LEVEL1;

run;

proc sort data=_crude_1;by PARM level1;run;

proc sort data=_adj_1;by PARM level1;run;

data reg_&out;

merge _crude_1 _adj_1;

by PARM level1;

run;

proc datasets;delete _crude: _adj:;quit;

%mend;

%out (diff_pain_d);

%out (diff_adl_d);

%out (diff_phq9_d);

/*ORAE outcomes

1. hosp_cpain: count of pain-related hospitalizations

2. ed_cpain: count of pain-related ED visit

3. OUD: count of opioid use disorder

4. OD: count of opioid overdose

key exposure: case (1: study group vs 0: comparison group)

other variables:

--log_time: log of follow-up days

--IPTW:

--bene_id_n: encrypted ID number represents each episode for each resident.

final_adv: final analytical dataset for pain-related hospitalization and ED visit outcomes (at the resident episode level)

final_adv_1: final analytical dataset for OUD and OD (at the resident episode level), excluding residents with OUD or OD at baseline.

*/

/*Poisson model*/

%macro adv_p (out);

/*crude*/

PROC GENMOD data=final_adv ;

CLASS bene_id_n case (ref='0') ;

MODEL &out=case /dist=poisson link=log offset=log_time;

ODS OUTPUT ParameterEstimates=_crude;

run;

data _crude_1;

retain Parameter Level1 RR RR_L RR_U Probz;

set _crude;

RR=round (exp (estimate), 0.01);

RR_L=round (exp (LOWERWaldCL), 0.01);

RR_U=round (exp (UPPERWaldCL), 0.01);

rename ProbChisq=Probz;

if Parameter='case';

keep RR: Parameter ProbChisq LEVEL1;

run;

/*weighted*/

PROC GENMOD data=final_adv;

CLASS bene_id_n case (ref='0') ;

MODEL &out=case /dist=poisson link=log offset=log_time;

WEIGHT IPTW;

ODS OUTPUT ParameterEstimates=_adj;

run;

data _adj_1;

retain Parameter Level1 RR_a RR_L_a RR_U_a Probz_a;

set _adj;

RR_a=round (exp (estimate), 0.01);

RR_L_a=round (exp (LOWERWaldCL), 0.01);

RR_U_a=round (exp (UPPERWaldCL), 0.01);

rename ProbChisq=Probz_a;

if Parameter='case';

keep RR: Parameter ProbChisq LEVEL1;

run;

proc sort data=_crude_1;by parameter level1;run;

proc sort data=_adj_1;by parameter level1;run;

data reg_&out;

merge _crude_1 _adj_1;

by parameter level1;

run;

proc datasets;delete _crude: _adj:; quit;

%mend;

%adv_p(hosp_cpain);

%adv_p(ed_cpain);

/*Negative Binomial model*/

%macro adv_nb (out);

/*crude*/

PROC GENMOD data=final_adv_1;

CLASS bene_id_n case (ref='0') ;

MODEL &out=case /dist=negbin link=log offset=log_time;

ODS OUTPUT ParameterEstimates=_crude;

run;

data _crude_1;

retain Parameter Level1 RR RR_L RR_U Probz;

set _crude;

RR=round (exp (estimate), 0.01);

RR_L=round (exp (LOWERWaldCL), 0.01);

RR_U=round (exp (UPPERWaldCL), 0.01);

rename ProbChisq=Probz;

if Parameter='case';

keep RR: Parameter ProbChisq LEVEL1;

run;

/*weighted*/

PROC GENMOD data=final_adv_1;

CLASS bene_id_n case (ref='0') ;

MODEL &out=case /dist=negbin link=log offset=log_time;

WEIGHT IPTW;

ODS OUTPUT ParameterEstimates=_adj;

run;

data _adj_1;

retain Parameter Level1 RR_a RR_L_a RR_U_a Probz_a;

set _adj;

RR_a=round (exp (estimate), 0.01);

RR_L_a=round (exp (LOWERWaldCL), 0.01);

RR_U_a=round (exp (UPPERWaldCL), 0.01);

rename ProbChisq=Probz_a;

if Parameter='case';

keep RR: Parameter ProbChisq LEVEL1;

run;

proc sort data=_crude_1;by parameter level1;run;

proc sort data=_adj_1;by parameter level1;run;

data reg_&out;

merge _crude_1 _adj_1;

by parameter level1;

run;

proc datasets;delete _crude: _adj:; quit;

%mend;

%adv_nb(OUD);

%adv_nb(OD);
