# Supplementary material for: Association of initiating CYP2D6-metabolized opioids with risks of adverse outcomes in older adults receiving antidepressants: A retrospective cohort study
Source: PLoS Med. 2025 Jun 2;22(6):e1004620. doi: 10.1371/journal.pmed.1004620 (PMC12129234; doi:10.1371/journal.pmed.1004620)
Supplement: S1 Table — (DOCX) [file pmed.1004620.s003.docx]

**S1 Table.** Medications of Interest Considered in the Study

| **Medication Class** | **Individual Medication** |
| --- | --- |
| **CYP2D6-metabolized Opioids** | codeine, hydrocodone, oxycodone, tramadol |
| **Antidepressants** |  |
| CYP2D6 Inhibitors | SSRI: fluoxetine, paroxetine,  SNRI: duloxetine,  TCA: doxepin,  Other: bupropion |
| CYP2D6 Neutrals | SSRI: sertraline, citalopram, escitalopram, fluvoxamine  SNRI: desvenlafaxine, levomilnacipran, milnacipran, venlafaxine  TCA: amitriptyline, amoxapine, clomipramine, desipramine, maprotiline, nortriptyline, protriptyline, imipramine, trimipramine  Others: mirtazapine, nefazodone, trazodone, vilazodone, phenelzine, tranylcypromine, isocarboxazid, vortioxetine |
| **Nonopioids** |  |
| Analgesics and antipyretics | acetaminophen, salicylamide, sodium thiosalicylate, ziconotide |
| NSAIDs | celecoxib, diclofenac potassium/sodium, etodolac, fenoprofen, flurbiprofen, ibuprofen, indomethacin, ketoprofen, ketorolac, meclofenamate, mefenamic acid, meloxicam, nabumetone, naproxen, oxaprozin, piroxicam, sulindac, tolmetin |
| Salicylates | Aspirin, diflunisal, choline magnesium trisalicylate, magnesium salicylate, and salsalate |
| **Adjuvant analgesics** |  |
| SNRI antidepressants | duloxetine, venlafaxine, desvenlafaxine, milnacipran, levomilnacipran |
| TCA antidepressants | amitriptyline, clomipramine, imipramine, doxepin, trimipramine, amoxapine, maprotiline, nortriptyline, desipramine, protriptyline, |
| Anticonvulsants (for pain treatment) | carbamazepine, fosphenytoin, gabapentin, lacosamide, lamotrigine, levetiracetam, oxcarbazepine, phenytoin, pregabalin, topiramate, and valproate |
| Skeletal muscle relaxants | baclofen, carisoprodol, chlorzoxazone, cyclobenzaprine, dantrolene, diazepam, metaxalone, methocarbamol, orphenadrine, tizanidine |
| **Other CNS Medications** |  |
| Benzodiazepines | alprazolam, estazolam, lorazepam, oxazepam, temazepam, triazolam, midazolam, chlordiazepoxide, clobazam, clonazepam, clorazepate, diazepam, prazepam, flurazepam, quazepam. |
| Antipsychotics | acetophenazine, chlorpromazine, droperidol, fluphenazine, haloperidol, loxapine, mesoridazine, molindone, perphenazine, pimozide, prochlorperazine, thioridazine, thiothixene, trifluoperazine, aripiprazole, asenapine, brexpiprazole, cariprazine, clozapine, iloperidone, fanapt, lurasidone, olanzapine, paliperidone, primavaserin, quetiapine, risperidone, ziprasidone. |
| Sedative-hypnotics | buspirone, meprobamate, eszopiclone, zaleplon, zolpidem, suvorexant, hydroxyzine, diphenhydramine, ramelteon, tasimelteon, amobarbital, butabarbital, pentobarbital, secobarbital, phenobarbital, mephobarbital, chloral hydrate, lemborexant |
| Anticonvulsants (excluding those for pain treatment) | [acetazolamide](https://www.medicines.org.uk/emc/search?q=Acetazolamide), brivaracetam, cenobamate, eslicarbazepine acetate, ethadione, ethotoin, ethosuximide, ezogabine, felbamate, fenfluramine, mephenytoin, methohexital, methsuximide, magnesium sulfate, perampanel, paramethadione, phenobarbital, primidone, rufinamide, stiripentol, sultiame, tiagabine, trimethadione, vigabatrin, zonisamide |

Abbreviations: NSAID, nonsteroidal anti-inflammatory; SSRI, selective serotonin reuptake inhibitor; SNRI, serotonin and norepinephrine reuptake inhibitor; CNS, central nervous system; TCA, tricyclic antidepressants
