# Supplementary material for: Association of initiating CYP2D6-metabolized opioids with risks of adverse outcomes in older adults receiving antidepressants: A retrospective cohort study
Source: PLoS Med. 2025 Jun 2;22(6):e1004620. doi: 10.1371/journal.pmed.1004620 (PMC12129234; doi:10.1371/journal.pmed.1004620)
Supplement: S2 Table — (DOCX) [file pmed.1004620.s004.docx]

**S2 Table**. *ICD-9-CM, ICD-10-CM,* or Procedure Codes for Disease Conditions and Service Care Considered in the Study

| **Disease, Condition, or Service Care** | ***ICD-9-CM, ICD-10-CM,* or Procedure Code** | **Algorithm** |
| --- | --- | --- |
| **Exclusion criteria** |  |  |
| Cancer diagnosis | CCS11-CCS43 | HCUP CCS for *ICD-9-CM or ICD-10-CM* |
| Hospice care | Admission date of hospice claims | At least 1 hospice claim in any diagnostic position |
| Palliative Care | DX: V 66.7  Provide specialty code: 17 | At least 1 inpatient, SNF, HHA, HOP, carrier, or DME claim with disease code in any diagnostic position; or at least 1 inpatient, SNF, HHA, HOP with provider specialty code |
| **ORAE outcomes** |  |  |
| Pain-related hospitalization | Admission to inpatient with a primary or secondary diagnosis of a chronic pain condition (see below) | Inpatient claims |
| Pain-related ED | Admission to ED with a primary or secondary diagnosis of a chronic pain condition (see below) | Inpatient claims |
| Opioid use disorder | ICD-9 codes:  304.00 (Opioid type dependence – unspecified)  304.01 (Opioid type dependence – continuous)  304.02 (Opioid type dependence – episodic)  304.70 (Combinations of opioid-type drug with any other – unspecified)  304.71 (Combinations of opioid type drug with any other – continuous)  304.72 (Combinations of opioid type drug with any other – episodic)  305.50 (Opioid abuse – unspecified)  305.51 (Opioid abuse – continuous)  305.52 (Opioid abuse – episodic)  ICD-10 codes:  F11.xx, excluding codes that indicated “in remission” or “subsequent encounter to identify an incident event. | At least 1 inpatient, SNF, HHA, or carrier claim with disease code in any diagnostic position |
| Opioid overdose | ICD-9 codes:  965.00 (Poisoning – opium (alkaloids), unspecified)  965.01 (Poisoning – heroin)  965.02 (Poisoning – methadone)  965.09 (Poisoning – opiates and related narcotics, other)  E850.0 (Accidental poisoning by heroin)  E850.1 (Accidental poisoning by methadone)  E850.2 (Accidental poisoning by other opiates and related narcotics)  ICD-10 codes:  T400X1- T400X5, T401X1-T401X4, T402X1-T403X5, T404X1-T404X5, T40601-T40605, T40691-T40695, excluding codes that indicated “in remission” or “subsequent encounter to identify an incident event. | At least 1 inpatient, SNF, HHA, or carrier claim with disease code in any diagnostic position |
| **Clinical conditions for which antidepressants are prescribed** |  |  |
| Mental disorder | Adjustment disorder (CCS 650), anxiety disorders (CCS 651), attention-deficit, conduct, and disruptive behavior disorders (CCS 652), impulse control disorders (CCS 656), mood disorders (CCS 657), personality disorders (CCS 658), schizophrenia and other psychotic disorders (CCS 659), Miscellaneous mental health disorders (CCS 670) | HCUP CCS for *ICD-9-CM or ICD-10-CM* |
| Sleep disorder | 291.8x, 307.4x, 327x, 333.94, 347.x, 780.5x, F51.x, G25.9, G47.X, R06.81 | At least 1 inpatient, SNF, HHA, HOP, carrier, or DME claim with disease code in any diagnostic position |
| Behavioral symptoms of dementia | 290.x, 293.x, 294.x, 297.x, 298.x, 307.9, 312.9, 780.1, 780.97, 310.X, 331.x, F01.x (excluding [F01.50](https://www.icd10data.com/ICD10CM/Codes/F01-F99/F01-F09/F01-/F01.50), [F01.A0](https://www.icd10data.com/ICD10CM/Codes/F01-F99/F01-F09/F01-/F01.A0), [F01.B0](https://www.icd10data.com/ICD10CM/Codes/F01-F99/F01-F09/F01-/F01.B0), [F01.C0](https://www.icd10data.com/ICD10CM/Codes/F01-F99/F01-F09/F01-/F01.C0)), F02.x (excluding [F02.80](https://www.icd10data.com/ICD10CM/Codes/F01-F99/F01-F09/F02-/F02.80), [F02.A0](https://www.icd10data.com/ICD10CM/Codes/F01-F99/F01-F09/F02-/F02.A0),  [F02.B0](https://www.icd10data.com/ICD10CM/Codes/F01-F99/F01-F09/F02-/F02.B0),  [F02.C0](https://www.icd10data.com/ICD10CM/Codes/F01-F99/F01-F09/F02-/F02.C0)), F03.x (excluding [F03.90](https://www.icd10data.com/ICD10CM/Codes/F01-F99/F01-F09/F03-/F03.90), [F03.B0](https://www.icd10data.com/ICD10CM/Codes/F01-F99/F01-F09/F03-/F03.B0),  [F03.A0](https://www.icd10data.com/ICD10CM/Codes/F01-F99/F01-F09/F03-/F03.A0),  [F03.C0](https://www.icd10data.com/ICD10CM/Codes/F01-F99/F01-F09/F03-/F03.C0)), F05, F06.x, F22-F24, F28-F29, F91.9, F94.2, R41.82. R44.0, R44.2, RR44.3, R45.1 |  |
| **Clinical condition** |  |  |
| Tobacco use disorder | 305.1, 649.0x, 989.84, F17.x, O99.33x, T65.21xA, Z72.0 | At least 1 inpatient, SNF, HHA, HOP, carrier, or DME claim with disease code in any diagnostic position |
| Alcohol use disorder | 291.x, 303.x, 305.0x, 357.5, 425.5, 535.3x, 571.0-571.3, 760.71, 980.0, V65.42, V79.1, E860.0, F10.x (excluding F10.11, F10.13, F10.21, F10.93), G62.1, I42.6, K29.2x, K70.x, P04.3, Q86.0, T51.0XxA, Z71.4x |  |
| Chronic pain |  |  |
| Musculoskeletal | 274.x, 710.x-729.x (exclude 723.4, 724.3, 724.4, 729.1, 729.2)  A18.01-A18.02, A52.16, D48.1, E08.61x, E09.61x, E10.61x, E11.61x, E13.61x, M00-M02, M04.02-M04.09, M05-M19, M1A, M20.10, M21.61-M21.62, M22-M25, M32-M36, M43.2-M43.8X9, M45-M48, M49.80, M50, M51, M53, M54, M60.0-M60.2, M61-M63, M65-M67, M70-M72, M75-M77, M79, M96.1, M99.2-M99.7, N20.0, Q68.6, R25.2, R26.2, R29.8x | At least 1 inpatient, SNF, HHA, HOP, or carrier claim with disease code in any diagnostic position |
| Neuropathic | 053.1x, 249.6, 250.6, 307.89, 336.x, 337.x, 338.0, 340, 350.x, 351.x, 352.1, 353.x-355.x, 357.1, 357.2-357.4, 357.8, 357.9, 723.4, 724.3, 724.4, 729.1, 729.2,  A52.15, B02 (exclude B02.1), EXX.4, EXX.610, EXX.65 (where X in “08”-“13”), E10.4, F45.42, G13.0, G13.1, G32.0, G35, G50- G52.1, G54-G59, G61.8, G61.9, G62.8, G62.9, G63-G65, G89.0, G90.0, G90.5, G95, G99.0-G99.2, M05.5, M54.13-M54.18, M54.3, M54.4, M60.8, M60.9, M79.1, M79.2, M79.7 |  |
| Idiopathic | 338.2, 338.4, 780.96, G89, R52 |  |
| Diabetes | Diabetes mellitus without complication (CCS 49), diabetes mellitus with complications (CCS 50) | HCUP CCS for *ICD-9-CM or ICD-10-CM* |
| Cardiovascular disease | Heart valve disorders (CCS 96), coronary atherosclerosis and other heart disease (CCS 101), pulmonary heart disease (CCS 103), cardiac dysrhythmias (CCS 106), congestive heart failure; nonhypertensive (CCS 108), acute cerebrovascular disease (CCS 109), occlusion or stenosis of precerebral arteries (CCS 110), other and ill-defined cerebrovascular disease (CCS 111), peripheral and visceral atherosclerosis (CCS 114) |  |
| Hypertension | essential hypertension (CCS 98), hypertension with complications, and secondary hypertension (CCS 99) |  |
| Pulmonary condition | Pneumonia (except that caused by tuberculosis or sexually transmitted disease) (CCS 122), acute bronchitis (CCS 125), other upper respiratory infections (CCS 126), chronic obstructive pulmonary disease and bronchiectasis (CCS 127), asthma (CCS 128), pleurisy; pneumothorax; pulmonary collapse (CCS 130), respiratory failure; insufficiency; arrest (CCS 131), other lower respiratory disease (CCS 133) |  |
| Kidney disease | Nephritis; nephritis; renal sclerosis (CCS 156), acute and unspecified renal failure (CCS 157), chronic kidney disease (CCS 158), other diseases of kidney and ureters (CCS 161) |  |
| Gastrointestinal tract disorder | Gastrointestinal hemorrhage (CCS 153), other gastrointestinal disorders (CCS 155), digestive congenital anomalies (CCS 214) |  |
| Liver disease | Liver diseases (CCS 151) |  |
| Injury | Pathological fracture (CCS 207), fracture of neck of femur (hip) (CCS 226), skull and face fractures (CCS 228), fracture of upper limb (CCS 229), fracture of lower limb (CCS 230), other fractures (CCS 231), e-codes: fall (CCS 2603). |  |
| Neurodegenerative disorder | Parkinson's disease (CCS 79), other hereditary and degenerative nervous system conditions (CCS 81) |  |
| Seizure | Epilepsy; convulsions (ccs 83) |  |
| Drug use disorder | Chronic Condition Data Warehouse, Drug Use Disorder Algorithm^1^ | At least 1 inpatient, SNF, HHA, HOP, carrier, or DME claim with disease code |
| Psychotic and disruptive behaviors | Presence or absence of any of the following symptoms: psychotic (delusions or hallucinations) and disruptive behaviors (rejection of care, or physical, verbal, or other aggressive behaviors) | MDS 3.0 |
| *Procedures* |  |  |
| Medical procedures and therapies for chronic pain management | 20550-20552, 20560, 20561, 28899, 20999, 62281, 62324, 62325, 64405, 64408, 64415, 64417, 64418, 64420, 64421, 64425, 64430, 64435, 64445-64451, 64461-64463, 64505, 64510, 64517, 64520, 64530, 64620, 64632, 64660, 64999, 64450, 76881, 76882, 76942, 76999, 90901, 90912, 90913, 92506, 97001-97004, 97010, 97012, 97016, 97018, 97022, 97024, 97026, 97028, 97032-97036, 97039, 97110, 97112, 97113, 97116, 97124, 97139, 97140, 97150, 97530, 97542, 97597, 97598, 97760, 97799, 97610, 97161-97168, 97810, 97811, 97813, 97814, G0281, G0282, G0283, G0329, 0019T, 98940, 98941, 98942, 98943 | At least 1 inpatient, SNF, HHA, HOP, carrier, or DME claim with disease code |

Abbreviations: CCS, Clinical Classification Software; DME, Duration Medical Equipment; ED, emergency department; HCUP, Healthcare Cost and Utilization Project; HHA, home health agency; HOP, hospital outpatient; ORAE, opioid-related adverse events; *ICD-9-CM and ICD-10-CM*, *International Classification of Diseases, Ninth or Tenth Revision, Clinical Modification*; SNF, skilled nursing facility. ^1^https://www2.ccwdata.org/web/guest/condition-categories-other
