# Supplementary material for: Association of initiating CYP2D6-metabolized opioids with risks of adverse outcomes in older adults receiving antidepressants: A retrospective cohort study
Source: PLoS Med. 2025 Jun 2;22(6):e1004620. doi: 10.1371/journal.pmed.1004620 (PMC12129234; doi:10.1371/journal.pmed.1004620)
Supplement: S3 Table — (DOCX) [file pmed.1004620.s005.docx]

**S3 Table**. Study Covariates, Definitions, and Measurement Sources and Windows

| **Covariate** | **Measurement** | **Measurement window** | **Data source** |
| --- | --- | --- | --- |
| **Demographic characteristics** |  |  |  |
| Age | Classified into 3 age groups: 65-74, 75-84, and ≥85 years | On cohort entry date | Medicare Beneficiary Summary File |
| Sex | Male or female | On cohort entry date | Medicare Beneficiary Summary File |
| Race/ethnicity | Measured based on the Medicare-Research Triangle Institute race code and grouped into 3 groups: White, Black, and other (including Hispanic, Asian, Pacific Islander, and Native American individuals) | On cohort entry date | Medicare Beneficiary Summary File |
| US geographic region | Northeast, Midwest, West, and South | On cohort entry date | Medicare Beneficiary Summary File |
| Receipt of low-income subsidy (LIS) | LIS receipt was defined as having at least 6 months of receiving LIS; otherwise, non-LIS receipt | 6 months before cohort entry date | Medicare Beneficiary Summary File |
| **Health Status** |  |  |  |
| Body mass index | MDS-documented weight in kilograms divided by the MDS-documented height in meters squared and categorized as underweight (BMI <18.5), normal (BMI 18.5-24.9), overweight (BMI 25.0-29.9), and obese (BMI ≥30) | 6 months before cohort entry date | MDS 3.0 |
| Tobacco or alcohol use disorder | Presence or absence of diagnosis | 6 months before cohort entry date | Medicare Parts A and B |
| **Clinical condition** |  |  |  |
| Chronic pain | Presence or absence of diagnosis | 6 months before cohort entry date | Medicare Parts A and B |
| Musculoskeletal | Presence or absence of diagnosis | 6 months before cohort entry date | Medicare Parts A and B |
| Neuropathic | Presence or absence of diagnosis | 6 months before cohort entry date | Medicare Parts A and B |
| Idiopathic | Presence or absence of diagnosis | 6 months before cohort entry date | Medicare Parts A and B |
| Mental health disorder | Presence or absence of diagnosis | 6 months before cohort entry date | Medicare Parts A and B |
| Sleep disorder | Presence or absence of diagnosis | 6 months before cohort entry date | Medicare Parts A and B |
| Behavioral symptoms of dementia | Presence or absence of diagnosis | 6 months before cohort entry date | Medicare Parts A and B |
| Hypertension | Presence or absence of diagnosis | 6 months before cohort entry date | Medicare Parts A and B |
| Diabetes | Presence or absence of diagnosis | 6 months before cohort entry date | Medicare Parts A and B |
| Cardiovascular disease | Presence or absence of diagnosis | 6 months before cohort entry date | Medicare Parts A and B |
| Pulmonary condition | Presence or absence of diagnosis | 6 months before cohort entry date | Medicare Parts A and B |
| Gastrointestinal disease | Presence or absence of diagnosis | 6 months before cohort entry date | Medicare Parts A and B |
| Injury | Presence or absence of diagnosis | 6 months before cohort entry date | Medicare Parts A and B |
| Kidney disease | Presence or absence of diagnosis | 6 months before cohort entry date | Medicare Parts A and B |
| Liver disease | Presence or absence of diagnosis | 6 months before cohort entry date | Medicare Parts A and B |
| Neurodegenerative disease | Presence or absence of diagnosis | 6 months before cohort entry date | Medicare Parts A and B |
| Seizure | Presence or absence of diagnosis | 6 months before cohort entry date | Medicare Parts A and B |
| Drug use disorder | Presence or absence of diagnosis | 6 months before cohort entry date | Medicare Parts A and B |
| Total number of comorbidities | Sum of the number of AHRQ CCS categories excluding aforementioned pain and clinical conditions | 6 months before cohort entry date | Medicare Parts A and B |
| **Pain management** |  |  |  |
| Use of drug or nondrug pain intervention | Yes vs. no | 6 months before cohort entry date | MDS 3.0 |
| Receipt of procedure or therapy for chronic pain management | Presence or absence of CPT codes | 6 months before cohort entry date | Medicare Parts A and B |
| Use of adjuvant analgesic | Yes vs. no | 6 months before cohort entry date | Medicare Part D |
| Use of prescription nonopioid | Yes vs. no | 6 months before cohort entry date | Medicare Part D |
| Use of PRN pain medication | Yes vs. no | 6 months before cohort entry date | MDS 3.0 |
| **Medication use** |  |  |  |
| Use of other CNS medication | Yes vs. no | 6 months before cohort entry date | Medicare Part D |
| Polypharmacy | Yes vs. no | 6 months before cohort entry date | Medicare Part D |
| Use of other CYP2D6 inhibitor | Yes vs. no | 6 months before cohort entry date | Medicare Part D |
| Use of other CYP3A4 inhibitor | Yes vs. no | 6 months before cohort entry date | Medicare Part D |
| Use of other CYP2D6 inducer | Yes vs. no | 6 months before cohort entry date | Medicare Part D |
| **Duration of antidepressant use** | Mean (SD) | 6 months before cohort entry date | Medicare Part D |
| **Baseline cognitive function** | Normal (BIMS, 13-15), mild (BIMS: 8-12 or CPS: 0-2), moderate (BIMS: 0-7 or CPS: 3-4), severe (CPS: 5-6) cognitive function. Note: patients with severe cognitive function at baseline were excluded | 6 months before cohort entry date | MDS 3.0 |
| **Baseline physical function** | No (ADL score ≤9), mild (10 ≤ ADL ≤18), moderate (19 ≤ ADL ≤ 27), or severe (ADL ≥28) | 6 months before cohort entry date | MDS 3.0 |
| **Baseline depressive symptoms** | No (PHQ-9 score ≤4), mild (5 ≤ PHQ-9 ≤ 9), moderate (10 ≤ PHQ-9 ≤ 14), or severe (PHQ-9 ≥15) | 6 months before cohort entry date | MDS 3.0 |
| **Baseline pain** | No, mild, moderate, or severe | 6 months before cohort entry date | MDS 3.0 |
| **Nursing home characteristic** |  |  |  |
| Total number of beds | Mean (SD) | On cohort entry date | MDS 3.0 linked to the LTCFocus data sets |
| Chain membership | Yes vs. no | On cohort entry date | MDS 3.0 linked to the LTCFocus data sets |
| Profit status | Yes vs. no | On cohort entry date | MDS 3.0 linked to the LTCFocus data sets |
| Any special care unit | Yes vs. no | On cohort entry date | MDS 3.0 linked to the LTCFocus data sets |
| Geography | Metropolitan (RUCC=1-3), micropolitan (RUCC=4-7), or rural (RUCC=8-9) | On cohort entry date | MDS 3.0 linked to the LTCFocus data sets and 2013 RUCC data |
| **Year of cohort entry** | Year in which an individual entered the cohort included 2011 (reference), 2012, 2013, 2014, 2015, 2016, 2017, 2018, 2019, and 2020 | On cohort entry date | Medicare Part D |

Abbreviations: ADL, activities of daily living; AHRQ CCS, Agency for Healthcare Research and Quality Clinical Classifications Software; BIMS, Brief Interview for Mental Status; BMI, body mass index; CYP, cytochrome P450; CNS, central nervous system; CPS, Cognitive Performance Scale; CPT, Current Procedural Terminology; MME, morphine milligram equivalents; PHQ-9, Patient Health Questionnaire-9; PRN, as needed; MDS, Minimum Data Set; RUCC, rural-urban continuum codes, SD, standard deviation.
