# Supplementary material for: Association of initiating CYP2D6-metabolized opioids with risks of adverse outcomes in older adults receiving antidepressants: A retrospective cohort study
Source: PLoS Med. 2025 Jun 2;22(6):e1004620. doi: 10.1371/journal.pmed.1004620 (PMC12129234; doi:10.1371/journal.pmed.1004620)
Supplement: S4 Table — (DOCX) [file pmed.1004620.s006.docx]

**S5 Table.** Quarterly Associations of Concomitant Use of Antidepressants with CYP2D6-Metabolized Opioids With Clinical Worsening Outcomes From Baseline to Follow-Up

|  | **CYP2D6-Metabolized Opioids Concomitantly Used with CYP2D6-inhibiting ADs (vs. CYP2D6-neutral ADs)** | | | | **Interaction of quarter time with concomitant opioid-AD use** | |
| --- | --- | --- | --- | --- | --- | --- |
| **Outcome by Quarters ^a^** | **Crude RR**  **(95% CI)** | ***P* value** | **Adjusted RR^b^**  **(95% CI)** | ***P* value** | **Adjusted RR^b^**  **(95% CI)** | ***P* value** |
| ***Worsening pain*** |  |  |  |  |  |  |
| Quarter 1 | 1.07 (1.05, 1.10) | <.001 | 1.03 (1.00, 1.05) | .08 | 1.01 (0.99, 1.02) | .40 |
| Quarter 2 | 1.14 (1.11, 1.17) | <.001 | 1.06 (1.02, 1.09) | <.001 |  |  |
| Quarter 3 | 1.15 (1.11, 1.18) | <.001 | 1.09 (1.05, 1.13) | <.001 |  |  |
| Quarter 4 | 1.11 (1.07, 1.14) | <.001 | 1.02(0.99, 1.06) | .22 |  |  |
| ***Worsening physical function*** | |  |  |  |  |  |
| Quarter 1 | 0.97 (0.96, 0.99) | <.001 | 0.99 (0.98, 1.01) | .44 | 1.00 (0.99, 1.01) | .49 |
| Quarter 2 | 0.97 (0.96, 0.99) | <.001 | 0.99 (0.98, 1.01) | .41 |  |  |
| Quarter 3 | 0.97 (0.96, 0.99) | <.001 | 1.00 (0.98, 1.01) | .81 |  |  |
| Quarter 4 | 0.98 (0.97, 0.99) | .002 | 1.00 (0.99, 1.01) | .98 |  |  |
| ***Worsening depressive symptoms*** | |  |  |  |  |  |
| Quarter 1 | 0.99 (0.97, 1.02) | .47 | 1.01 (0.99, 1.04) | .39 | 0.99 (0.98, 1.01) | .33 |
| Quarter 2 | 0.99 (0.97, 1.02) | .61 | 1.01 (0.99, 1.04) | .33 |  |  |
| Quarter 3 | 1.00 (0.97, 1.02) | .91 | 1.02 (0.99, 1.04) | .28 |  |  |
| Quarter 4 | 0.98 (0.95, 1.00) | .07 | 0.99 (0.96, 1.02) | .55 |  |  |

Abbreviations: AD, antidepressants; CYP, cytochrome P450; RR, rate ratio.

^a^ Logistic regression model with a generalized estimating equation that adjusted for baseline covariates via the inverse probability of treatment weighting and quarter (time) as covariates for clinical outcomes.
