# Supplementary material for: Association of initiating CYP2D6-metabolized opioids with risks of adverse outcomes in older adults receiving antidepressants: A retrospective cohort study
Source: PLoS Med. 2025 Jun 2;22(6):e1004620. doi: 10.1371/journal.pmed.1004620 (PMC12129234; doi:10.1371/journal.pmed.1004620)
Supplement: S5 Table — Quarterly associations of concomitant use of CYP2D6-metabolized opioids and antidepressants with clinical worsening outcomes from baseline to follow-up. (DOCX) [file pmed.1004620.s007.docx]

**S6 Table.** Unadjusted and Adjusted Associations of Concomitant Use of CYP2D6-Metabolized Opioids and Antidepressants with Clinical Worsening and Opioid-Related Adverse Outcomes, Stratified By Specific CYP2D6-Metabolized Opioid

|  | **CYP2D6-metabolized opioids concomitantly used with CYP2D6-inhibiting ADs (vs. CYP2D6-neutral ADs)** | | | | | | | | | | | |
| --- | --- | --- | --- | --- | --- | --- | --- | --- | --- | --- | --- | --- |
|  | **Residents receiving tramadol (n=50483)** | | | | **Residents receiving hydrocodone (n=45627)** | | | | **Residents receiving oxycodone (n=11472)** | | | |
| **Clinical Outcomes** ^a^ | **Crude RR (95% CI)** | **P-value** | **Adjusted RR**^b^  **(95% CI)** | **P-value** | **Crude OR (95% CI)** | **P-value** | **Adjusted RR**^b^  **(95% CI)** | **P-value** | **Crude OR (95% CI)** | **P-value** | **Adjusted RR**^b^  **(95% CI)** | **P-value** |
| Worsening pain | 1.22 (1.08, 1.15) | <.001 | 1.04 (1.00, 1.07) | .04 | 1.09 (1.06, 1.12) | <.001 | 1.04 (1.01, 1.08) | .01 | 1.19 (1.12, 1.26) | <.001 | 1.12 (1.05, 1.19) | <.001 |
| Worsening physical function | 0.98 (0.97, 0.99) | .03 | 1.01 (0.99, 1.02) | .33 | 0.97 (0.95, 0.98) | <.001 | 0.99 (0.97, 1.00) | .08 | 0.98 (0.95, 1.01) | .15 | 1.00 (0.97, 1.04) | .90 |
| Worsening depression | 1.02 (0.99, 1.04) | .28 | 1.02 (0.99, 1.05) | .25 | 0.97 (0.94, 0.99) | .04 | 1.00 (0.97, 1.03) | .95 | 0.97 (0.92, 1.03) | .36 | 1.01 (0.94, 1.08) | .82 |
|  | **Residents with Tramadol (n=61039)** | | | | **Residents with hydrocodone (n=54681)** | | | | **Residents with oxycodone (n=13934)** | | | |
| **Adverse outcomes** | **Crude IRR (95% CI)** | **P-value** | **Adjusted IRR**^c^ **(95% CI)** | **P-value** | **Crude IRR (95% CI)** | **P-value** | **Adjusted IRR**^c^ **(95% CI)** | **P-value** | **Crude IRR (95% CI)** | **P-value** | **Adjusted IRR**^c^ **(95% CI)** | **P-value** |
| Pain-related hospitalization | 1.42 (1.17, 1.73) | <.001 | 1.10 (1.03, 1.24) | .03 | 1.49 (1.22, 1.81) | <.001 | 1.26 (1.11, 1.42) | <.001 | 1.02 (0.68, 1.53) | .92 | 0.83 (0.65, 1.07) | .15 |
| Pain-related ED visit | 1.47 (1.13, 1.90) | .004 | 1.17 (1.01, 1.37) | .04 | 1.50 (1.14, 1.97) | .004 | 1.32 (1.11, 1.56) | .002 | 0.89 (0.49, 1.61) | .70 | 0.90 (0.64, 1.26) | .53 |
| Opioid use disorder ^e^ | 1.20 (0.69, 2.37) | .61 | 1.12 (0.66, 1.90) | .68 | 1.22 (0.64, 2.33) | .54 | 1.10 (0.66, 1.82) | .72 | 0.85 (0.30, 2.42) | .76 | 0.52 (0.23, 1.17) | .12 |
| Opioid overdose ^e^ | 1.17 (0.73, 1.89) | .51 | 0.87 (0.56, 1.35) | .54 | 1.50 (0.92, 2.45) | .10 | 1.34 (0.88, 2.05) | .18 | 1.72 (0.84, 3.52) | .14 | 1.68 (0.88, 3.19) | .12 |

**S6 Table (Continued).** Unadjusted and Adjusted Associations of Concomitant Use of CYP2D6-Metabolized Opioids and Antidepressants With Clinical Worsening and Opioid-Related Adverse Outcomes, Stratified By Specific CYP2D6-Metabolized Opioids

|  | **CYP2D6-metabolized opioids concomitantly used with CYP2D6-inhibiting ADs (vs. CYP2D6-neutral ADs)** | | | | |
| --- | --- | --- | --- | --- | --- |
|  | **Residents receiving codeine (n=7082)** | | | | |
| **Clinical Outcomes** ^a^ | **Crude RR**  **(95% CI)** | **P-value** | **Adjusted RR**^b^  **(95% CI)** | **P-value** |  |
| Worsening pain | 1.08 (0.99-1.17) | .08 | 1.04 (0.95-1.14) | .40 |  |
| Worsening physical function | 0.97 (0.94-1.01) | .14 | 0.98 (0.94-1.02) | .38 |  |
| Worsening depression | 0.98 (0.91-1.05) | .53 | 0.99 (0.92-1.08) | .89 |  |
|  | **Residents receiving codeine (n=8342)** | | | |  |
| **Adverse outcomes** | **Crude IRR**  **(95% CI)** | **P-value** | **Adjusted IRR**^c^  **(95% CI)** | **P-value** |  |
| Pain-related hospitalization | 0.95 (0.55, 1.63) | .85 | 1.02 (0.77, 1.37) | .88 |  |
| Pain-related ED visit | 0.68 (0.31, 1.51) | .34 | 0.82 (0.55, 1.22) | .33 |  |
| Opioid use disorder ^d^ | --^e^ | --^e^ | --^e^ | --^e^ |  |
| Opioid overdose ^d^ | --^e^ | --^e^ | --^e^ | --^e^ |  |

Abbreviations: AD, antidepressants; CYP, cytochrome P450; RR; incidence rate ratio; MDS, Minimum Data Set; RR, rate ratio.

^a^ Clinical outcomes were measured in a subset that had at least one MDS 3.0 in follow-up.

^b^ A robust Poisson regression model with a generalized estimating equation that adjusted for baseline covariates via the inverse probability of treatment weighting and quarter (time) as covariates for clinical outcomes.

^c^ Poisson or negative binomial regression that adjusted for baseline covariates via the inverse probability of treatment weighting and total number of days in follow-up as an offset variable.

^d^ Restricted to the sample with no diagnosis of opioid use disorder or overdose at baseline.

^e^ No reports because of no or rare (<10) outcome events, leading to the model not converging or providing an unreliable estimate.
