# Supplementary material for: Association of initiating CYP2D6-metabolized opioids with risks of adverse outcomes in older adults receiving antidepressants: A retrospective cohort study
Source: PLoS Med. 2025 Jun 2;22(6):e1004620. doi: 10.1371/journal.pmed.1004620 (PMC12129234; doi:10.1371/journal.pmed.1004620)
Supplement: S7 Table — (DOCX) [file pmed.1004620.s009.docx]

**S7 Table**. Associations of Concomitant Use of Antidepressants and CYP2D6-Metabolized Opioids With Clinical Worsening and Opioid-Related Adverse Outcomes, Adjusting For Censoring Due to Death via Inverse Probability of Censoring Weighting

|  | **Concomitant Use of CYP2D6-inhibiting ADs (study group)**  **vs. CYP2D6-neutral ADs (comparison group) with CYP2D6-Metabolized Opioids** | | | |
| --- | --- | --- | --- | --- |
| **Clinical Outcomes** ^a^ | **Crude RR (95% CI)** | **P-value** | **Adjusted RR**^b^ **(95% CI)** | **P-value** |
| Worsening pain | 1.10 (1.08-1.12) | <.001 | 1.04 (1.02-1.06) | <.001 |
| Worsening physical function | 0.97 (0.96-0.98) | <.001 | 1.00 (0.99-1.01) | .54 |
| Worsening depression | 0.99 (0.97-1.00) | .14 | 1.01 (0.99-.1.03) | .62 |
| **Adverse outcomes** | **Crude IRR (95% CI)** | **P-value** | **Adjusted IRR**^c^ **(95% CI)** | **P-value** |
| Pain-related hospitalization | 1.36 (1.20, 1.55) | <.001 | 1.13 (1.05, 1.21) | .001 |
| Pain-related ED visit | 1.34 (1.13, 1.60) | <.001 | 1.16 (1.05, 1.28) | .003 |
| Opioid use disorder ^d^ | 1.35 (0.89, 2.03) | .16 | 1.18 (0.84, 1.64) | .34 |
| Opioid overdose ^d^ | 1.37 (1.01, 1.85) | .04 | 1.18 (0.91, 1.54) | .21 |

Abbreviations: AD, antidepressants; CYP, cytochrome P450; ED, emergency department; MDS, minimum data set; IRR, incidence rate ratio; RR, rate ratio.

^a^ Clinical outcomes were measured in a subset of the sample with at least one MDS 3.0 in follow-up.

^b^ Logistic regression model with a generalized estimating equation that adjusted for baseline covariates via the inverse probability of treatment weighting, censoring due to death via the inverse probability of censoring weighting, and quarter (time) as covariates for clinical outcomes.

^c^ Poisson or negative binomial regression that adjusted for baseline covariates via the inverse probability of treatment weighting, censoring due to death via the inverse probability of censoring weighting, and total number of days in follow-up as an offset variable.

^d^ Restricted to the sample with no diagnosis of opioid use disorder or overdose at baseline.
