# Supplementary material for: Association of initiating CYP2D6-metabolized opioids with risks of adverse outcomes in older adults receiving antidepressants: A retrospective cohort study
Source: PLoS Med. 2025 Jun 2;22(6):e1004620. doi: 10.1371/journal.pmed.1004620 (PMC12129234; doi:10.1371/journal.pmed.1004620)
Supplement: S8 Table — (DOCX) [file pmed.1004620.s010.docx]

**S8 Table**. Sensitivity Analysis of Including Eligible Residents Without Use of Other CYP2D6 Medications at Baseline

|  | **CYP2D6-Metabolized Opioids Concomitantly Used with CYP2D6-inhibiting ADs (vs. CYP2D6-neutral ADs)** | | | | |
| --- | --- | --- | --- | --- | --- |
|  | Number of Residents (n=104324) | | | |  |
| **Clinical Outcomes** ^a^ | **Crude RR**^b^ **(95% CI)** | **P-value** | **Adjusted RR**^b^ **(95% CI)** | **P-value** |  |
| Worsening pain | 1.11 (1.09-1.13) | <.001 | 1.05 (1.02-1.07) | <.001 |  |
| Worsening physical function | 0.97 (0.97-0.98) | <.001 | 1.00 (0.99-1.01) | 0.50 |  |
| Worsening depression | 0.99 (0.98-1.01) | 0.51 | 1.01 (0.99-1.03) | 0.26 |  |
|  | Number of Residents (n=122880) | | | |  |
| **Adverse outcomes** | **Crude IRR**^c^ **(95% CI)** | **P-value** | **Adjusted IRR**^c^ **(95% CI)** | **P-value** |  |
| Pain-related hospitalization | 1.36 (1.19, 1.55) | <.001 | 1.08 (1.00, 1.16) | .04 |  |
| Pain-related ED visit | 1.34 (1.12, 1.60) | <.001 | 1.13 (1.02, 1.26) | .02 |  |
| Opioid use disorder ^d^ | 1.39 (0.90, 2.13) | .13 | 1.04 (0.93, 1.16) | .50 |  |
| Opioid overdose ^d^ | 1.44 (1.06, 1.97) | .02 | 1.11 (0.97, 1.26) | .12 |  |

Abbreviations: AD, antidepressants; CYP, cytochrome P450; IRR; incidence rate ratio; RR, rate ratio.

^a^ A resident could contribute to more than one observation during the study period.

^b^ A robust Poisson regression model with a generalized estimating equation that adjusted for baseline covariates via the inverse probability of treatment weighting and quarter (time) as covariates for clinical outcomes.

^c^ Poisson or negative binomial regression that adjusted for baseline covariates via the inverse probability of treatment weighting and total number of days in follow-up as an offset variable

^d^ Restricted to the sample with no diagnosis of opioid use disorder or overdose at baseline.
