# Supplementary material for: Association of initiating CYP2D6-metabolized opioids with risks of adverse outcomes in older adults receiving antidepressants: A retrospective cohort study
Source: PLoS Med. 2025 Jun 2;22(6):e1004620. doi: 10.1371/journal.pmed.1004620 (PMC12129234; doi:10.1371/journal.pmed.1004620)
Supplement: S9 Table — Sensitivity analysis of including eligible residents’ first observation only. (DOCX) [file pmed.1004620.s011.docx]

**S9 Table**. Sensitivity Analysis of Including Eligible Residents’ First Observation Only

|  | **CYP2D6-Metabolized Opioids Concomitantly Used with CYP2D6-inhibiting ADs (vs. CYP2D6-neutral ADs)** | | | | |
| --- | --- | --- | --- | --- | --- |
|  | Number of Residents (n=108,127) | | | |  |
| **Clinical Outcomes** ^a^ | **Crude RR**^b^ **(95% CI)** | **P-value** | **Adjusted RR**^b^ **(95% CI)** | **P-value** |  |
| Worsening pain | 1.11 (1.08, 1.12) | <.001 | 1.04 (1.02, 1.06) | <.001 |  |
| Worsening physical function | 0.97 (0.96, 0.98) | <.001 | 1.00 (0.99, 1.01) | 0.50 |  |
| Worsening depression | 0.99 (0.97, 1.00) | 0.13 | 1.01 (0.99, 1.03) | 0.60 |  |
|  | Number of Residents (n=127,200) | | | |  |
| **Adverse outcomes** | **Crude IRR**^c^ **(95% CI)** | **P-value** | **Adjusted IRR**^c^ **(95% CI)** | **P-value** |  |
| Pain-related hospitalization | 1.38 (1.21, 1.58) | <.001 | 1.10 (1.02, 1.19) | .014 |  |
| Pain-related ED visit | 1.38 (1.15, 1.66) | <.001 | 1.15 (1.04, 1.28) | .007 |  |
| Opioid use disorder ^d^ | 1.35 (0.86, 2.11) | .19 | 1.14 (0.81, 1.61) | .44 |  |
| Opioid overdose ^d^ | 1.30 (0.94, 1.81) | .12 | 1.16 (0.87, 1.54) | .32 |  |

Abbreviations: AD, antidepressants; CYP, cytochrome P450; IRR; incidence rate ratio; RR, rate ratio.

^a^ A resident could contribute to more than one observation during the study period.

^b^ A robust Poisson regression model with a generalized estimating equation that adjusted for baseline covariates via the inverse probability of treatment weighting and quarter (time) as covariates for clinical outcomes.

^c^ Poisson or negative binomial regression that adjusted for baseline covariates via the inverse probability of treatment weighting and total number of days in follow-up as an offset variable

^d^ Restricted to the sample with no diagnosis of opioid use disorder or overdose at baseline.
