# Supplementary material for: Association of initiating CYP2D6-metabolized opioids with risks of adverse outcomes in older adults receiving antidepressants: A retrospective cohort study
Source: PLoS Med. 2025 Jun 2;22(6):e1004620. doi: 10.1371/journal.pmed.1004620 (PMC12129234; doi:10.1371/journal.pmed.1004620)
Supplement: Attachment — Submitted filename: Reviewers comments.docx [file pmed.1004620.s013.docx]

This large cohort study utilized nationwide Medicare data from the USA to compare clinical outcomes among older nursing home residents who initiated CYP2D6-metabolized opioids while already using CYP2D6-inhibiting versus CYP2D6-neutral antidepressants between 2010 and 2021. The authors followed individuals for one year and analyzed three distinct outcome groups: pain intensity, physical functioning, and depression. After adjusting for a wide range of potential confounders and covariates using inverse probability of treatment weighting, the study found that users of CYP2D6-inhibiting antidepressants were associated with worsened pain-related outcomes, including increased rates of pain-related hospitalizations and emergency department (ED) visits. However, no significant differences were observed between the groups in physical functioning or depressive symptoms.

This study is well-designed and structured, leveraging exceptionally large-scale data to address an important research question about the vulnerable older population. The authors effectively controlled for confounding variables using robust statistical methods and conducted a wide range of sensitivity analyses. The manuscript is well-written, with a balanced presentation of the relevant literature. Moreover, the conclusions are well-supported by the data and results. However, the current reviewer has outlined the following queries, requests for clarification, and suggested revisions to further enhance the manuscript's quality:

1. **Introduction**: While the authors discuss the potential mechanisms underlying interactions between these drug groups and their impact on outcomes to some extent, further elaboration on these mechanisms would strengthen the study's background and justification. This would help establish clearer hypotheses for the three outcomes: worsening depressive symptoms, impaired physical functioning, and increased pain intensity.
2. **Methods – Covariates**: Why was the calendar year not included as a covariate? Prescribing patterns for opioids and antidepressants, as well as clinical guidelines and care facility utilization, likely changed over time and could influence the outcomes.
3. **Sensitivity Analyses**: It would be valuable to perform an additional analysis restricting antidepressant users to those with a treatment indication of depression. Since antidepressants in older adults may also be prescribed for non-depressive indications (e.g., pain), excluding individuals with such indications could help clarify and rule out the possibility that worsening pain intensity was due to selection bias—specifically, the inclusion of individuals already using antidepressants for pain who may have experienced greater worsening in pain intensity.
4. If data on treatment indications for antidepressants and opioids are unavailable, this limitation should be acknowledged, as confounding by indication is a significant concern in observational research.
5. **Sensitivity Analyses – Clarification**: The sensitivity analyses section would benefit from further explanation of the purpose of each sensitivity analysis and what potential biases or issues they aim to address. This may not be immediately clear to all readers.
6. **Discussion**: In the first paragraph of the discussion section, the authors state that "CYP2D6 enzyme led to worsening pain control." The phrase "led to" implies causality, which is inappropriate in this context. The statement should be revised to avoid suggesting causation.
7. **Public Health Impact**: To enhance the discussion of public health implications, the authors could calculate an attributable fraction for the population. This would estimate how many excess cases of hospitalizations or ED visits could be prevented by prescribing CYP2D6-neutral antidepressants alongside CYP2D6-metabolized opioids.
8. **Limitations**: Regarding the first limitation, how large is the group of individuals whose prescriptions were self-paid or covered by non-Medicare programs? Including this information would provide additional context.
9. **Prescriber Information**: Is there any data on prescriber qualifications (e.g., general practitioners, psychiatrists, or other specialists)? If prescriber characteristics might influence drug selection and treatment outcomes, this limitation should be discussed.
10. **Tables**: The presentation of findings in Tables 2 and 3 could be improved by using forest plots, which would make the results more visually engaging and easier to interpret compared to the current monotone tabular format.
