# Supplementary material for: Association of initiating CYP2D6-metabolized opioids with risks of adverse outcomes in older adults receiving antidepressants: A retrospective cohort study
Source: PLoS Med. 2025 Jun 2;22(6):e1004620. doi: 10.1371/journal.pmed.1004620 (PMC12129234; doi:10.1371/journal.pmed.1004620)
Supplement: Attachment — Submitted filename: PlosMed Response_3.20.2025.docx [file pmed.1004620.s014.docx]

**Response to Editor’s and Reviewer’s comments on Manuscript ID: PMEDICINE-D-24-03810R1 (Due by Mar 27 2025 11:59 PM)**

| **Reviewer’s Comments** | **Our Response** |
| --- | --- |
| **Reviewer #1** |  |
| 1. [MINOR] I understand there is a lot to squeeze into the abstract but the fact all analyses are based on IPTW is important to know. I'd encourage the authors to find some way to identify this in either the abstract or, alternatively, the title. | We specified the use of inverse probability of treatment weighting for covariate adjustment in the abstract as follows:  “After covariate adjustment via the inverse probability of treatment weighting, use of CYP2D6-inhibiting (vs. CYP2D6-neutral) antidepressants…” **(Abstract, Page 3, Line 24)** |
| 2. What is the difference between a "nursing home" and a "skilled nursing facility" and what was the reasoning behind omitting patients with a stay at the latter? This excluded a substantial number of patients, and the reasoning isn't immediately clear to non-US readers. Did you consider sensitivity analyses including these patient (or separate analyses exclusively on these patients)? | A ‘nursing home’ provides care for various activities of daily living (eg., bathing, dressing, and eating) mainly for a long-term period (defined as > 100 nursing home days), although some may also provide care for a short-term period (defined as ≤100 nursing home days). The definition of short- vs long-term nursing home care using 100 days as a cut-off point is based on the Centers for Medicare and Medicaid Services (CMS) definition. Conversely, a ‘skilled nursing home facility” provides care only for a short-term period, with no long-term care services. It is noted that the vast majority (≥98%) of nursing homes or skilled nursing facilities in the US provide services for beneficiaries in Medicare and/or Medicaid, and thus CMS is the major payor.  The main reason for excluding individuals who received skilled nursing homes because all Medicare-covered services provided during this period are ***not*** available due to their prospective payment system policy, which is a single, fixed payment for a combined cost of eligible Medicare services for medical encounters and prescription data. In other words, there are no encounter claims records of Medicare Parts A and B available for capturing diagnosis of conditions nor records of Medicare prescription Part D data for capturing concomitant use of prescription opioids and antidepressants, the key exposure in our data. Similarly, we excluded individuals with hospital stays because, during this period, records for Medicare-covered services were *not* available due to the bundled payment policy.  As mentioned in our Method, the requirement of excluding individuals with skilled nursing home facilities at baseline is to ensure complete capture of baseline medical and prescription claims.  We have clarified this point as follows in Method:  “no hospital or skilled nursing facility stay, during which Medicare-covered services are unavailable due to CMS’s prospective payment system policy [13];” **(Method, Page 10, Lines 149-150)**  We have also acknowledged the limitation of excluding individuals with skilled nursing home facilities as follows in the Discussion”  “Finally, our results can be generalized only to older long-term NH residents.” **(Discussion, Page 21, Lines 438)** |
| 3. Also with respect to inclusion/exclusion, Fig 1 says ~70k patients were excluded because they did not have a "… diagnosis of chronic pain for CYP2D6-metabolised opioids during 1 month before cohort entry", but I did not see this in the methods. Suggest making this clearer in the body of the manuscript given the large number of excluded patients. | We thank the reviewer for noting this and have now included this exclusion criterion in the Method as follows:  “To minimize confounding by disease conditions, we restricted the study sample to those with a diagnosis of chronic pain for opioid therapy initiation within 1 month and conditions that are FDA-approved or off-label use for antidepressants within 6 months before the cohort entry.” **(Method, Study Sample, Page 9, Lines 137-140)** |
| 4. "Residents were followed up from cohort entry until the end of 1-year follow-up, Medicare disenrollment, death, NH discharge, or study end (December 31, 2021)." Two things. Firstly, suggest the distribution of these outcomes is reported. Secondly, why did the authors enrol patients in 2021 with the knowledge they would not have a full year of follow-up? That is, why not cease enrolling new participants at the end of 2020 to ensure all enrolled patients could have had a full year of follow-up? One risk here is the influence of unobserved confounders that vary over time. | We have reported the distribution of patients censored due to Medicare enrollment, death, and NH discharge as follows:  “Among the 127,200 eligible sample, 7.1% of residents were censored due to Medicare disenrollment, 11.8% due to NH discharge, and 17.5% due to death. We observed similar proportions of residents censored due to Medicare disenrollment (7.3% vs 7.2%) and NH discharge (11.5% vs 11.8%), but different proportions due to death (13.8% vs 17.9%) between the study and comparison group, which was adjusted via the inverse probability of censoring weighting (IPCW) as a sensitivity analysis*.”* **(Results, Page 15, Lines 239-298)**  We have specified that the enrollment ends in December 2020 as follows:  “The study sample included older (≥65 years) long-term residents who have CYP2D6-metabolized opioids initiated while already receiving antidepressants for at least 30 days between January 1, 2011 and December 31, 2020.” **(Method, Study Sample, Page 9, Lines 133-135)** |
| 5. "Residents were allowed to re-enter the cohort after the end of follow-up if they met the eligibility criteria." How did the authors account for this non-independence in their statistical analyses? | We thank the reviewer for the comment. We attempted to address the potential for non-independence within individuals who later entered the cohort (n=9860, 7.8% of the 127,200 individuals for analysis of ORAE outcomes; and n=5834, 5.4% of 108,127 individuals for analysis of clinical outcomes) using hierarchical regression models. Specifically, we used PROC GLIMMIX with individual beneficiary IDs in a Random Statement to account for repeated individual observations over time. However, the models **failed to converge** large due to the aforementioned, small proportions (7.8% and 5.4%) of our cohort samples who re-entered the cohort, with the vast majority of samples who entered the cohort only once.  Alternatively, to address the non-independence issue, we conducted a sensitivity analysis by including only the first individual observation, described as follows:  “Fifth, to address the potential non-independence within individual observations due to cohort re-entry, we included the first eligible observation of each study sample” **(Method, Statistical Analysis, Page 15, Lines 281-283)**  We also provided the results of this sensitive analysis as follows”  “The sensitivity analyses … and included only the first eligible resident observation (**S9 Table**) yielded results consistent with the main analysis for clinical and ORAE outcomes.” **(Results, Page 17, Lines 341-344)** |
| 6. Relatedly, I note that the authors incorporated some site-level covariates, but I don't think the site/patient/observation hierarchy was incorporated into the statistical modelling. Please clarify and consider the risk of site-level variation not adequately captured in the included site-level covariates. | We also attempted to account for the potential clustering effect across nursing home (NH) facilities (n=15,335 facilities for a total of 127,200 residents) in statistical modeling using hierarchical regression models. In particular, we used PROC GLIMMIX with NH facility IDs in a Random statement to account for the facility-level variation. However, the models **failed to converge** largely because 68% of the 15,335 NH facilities included less than 10 resident samples. Alternatively, we used the geography of NH location (classified into metropolitan, micropolitan, and rural areas) in the Random statement to ensure a sufficient number of residents within each sub-geographical NH location in the PROC GLIMMIX (hierarchical model). We showed the results from hierarchical models vs. previously reported results using general Poisson or negative binomial regression models for ORAE outcomes. \| **Adjusted IRR 95% CI comparing the study vs comparison group** \|; ORAE outcomes \| Hierarchical models \| General Poisson/Neg Binomial models; Pain-related hosp. \| 1.12 (1.04-1.22) \| 1.12 (1.04-1.21); Pain-related ED \| 1.17 (1.06-1.30) \| 1.17 (1.05-1.30); OUD^2^ \| 1.17 (0.85-1.63) \| 1.17 (0.84-1.63); OD^2^ \| 1.21(0.93-1.60) \| 1.21(0.93-1.59) ^1^ was generated using a Poisson or negative binomial model ^2^ restricted to samples without OUD or OD during the 6-month baseline. As indicated in the above table, the adjusted IRR estimates for OARE outcomes are identical with no changes between the two types of models, suggesting that facility-level variation does not affect our estimates. We also observed similar estimates for dichotomous clinical outcomes between the hierarchical models and general robust Poisson models. In this revision, we were asked by reviewers to provide the adjusted absolute risk of the study and comparison group and the adjusted risk difference between groups for the outcomes. To generate these absolute estimates, a SAS macro ([https://support.sas.com/kb/63/038.html](https://urldefense.com/v3/__https:/support.sas.com/kb/63/038.html__;!!KGKeukY!1GqMd4i2hJBmPhbzEGUg0ijvau7kV9yujeg3_ZavB2O1uPFAjKxkue9cRnU2buSl9CXOYf-K8w4QtEAciBRcBw$); Reference 33) was used, which was described as follows: “We also calculated the adjusted absolute risk of the study and treatment group and the adjusted risk difference (RD) between groups for each clinical and ORAE outcome using a SAS macro [33].” **(Method, Statistical Analysis, Page 14, Lines 267-269)** Please note that the SAS macro is the only feasible approach that can work for our data that are available through the CMS Virtual Research Data Center. The SAS macro can only work for general regression models, *not models with random effects (see above reference/limitations).* For this reason, we prefer using the general regression models, which provide similar estimates to those generated using hierarchical models. |
| 7. Please describe how the propensity score was calculated and include an appropriate reference. | We have detailed the calculation of the propensity score by citing an appropriate reference as follows:  “Propensity score was estimated using a logistic regression that modeled the probability of being assigned to the study vs comparison group as the dependent variable, and baseline covariates as the independent variables [29]. **(Method, Statistical Analysis, Pages14, Lines 252-254)** |
| 8. Please justify your choice of IPTW over other propensity score-based methods (such as propensity score matching) and include an appropriate reference. Note I am not suggesting the authors shouldn't have used IPTW, rather it is one option of several with different pros and cons so understanding the reasoning is important. | We have justified using IPTW over other propensity score approaches as follows:  “We used ITPW because of its advantages in retaining all study participants and reporting balance in baseline characteristics between the study and comparing group [30].” **(Method, Statistical Analysis, Page 14, Lines 255-257)** |
| 9. "For each clinical outcome, we used a modified Poisson regression…" How was it modified and why? I suspect you mean robust Poisson regression was used but this needs clarification and an appropriate reference. | The reviewer is correct that we used a robust Poisson regression, which is also called the modified Poisson first introduced by Zou [15033648]. To avoid confusion, we have changed it to a ‘robust Poisson regression’. The purpose of using the model is to generate estimates of relative risk, which is clarified as follows:  “For each clinical outcome, we used a robust Poisson regression model [31] to estimate the adjusted risk ratio (aRR) and its 95% confidence interval (CI) of a worsening outcome…” **(Method, Statistical Analysis, Page 14, Line 258)** |
| 10. "For ORAE count outcomes, we used a Poisson or negative binomial model…" How was this decision made? I suspect based on the presence of overdispersion but important to make clear. | We have clarified that the choice of a Poisson or negative model for count outcomes is based on the presence of overdispersion as follows:  “For ORAE count outcomes, we used a Poisson or negative binomial model based on the presence of overdispersion [32] …” **(Method, Statistical Analysis, Page 14, Line 264)** |
| 11. I don't understand why reference [20] was included. The cited literature examined zero-inflated mixture models which are not employed here. | We thank the reviewer for noting this error and have corrected it by citing a relevant reference [PMID:7501743, reference 32] that discussed regression analyses of count data using Poisson vs negative binomial models. |
| 12. Similarly, I don't understand why reference [21] was included. The cited literature examined IPCW but as a win ratio statistic which is not used here. Further clarity here is important - what problem are you trying to solve and why is your choice of approach the optimal solution? | The purpose of this sensitivity analysis is to account for censoring and competing risk of death, which differs between the study and treatment group (please see our response to Reviewer#1, Q4). Inverse probability of censoring weighting [IPCW] is one of the commonly used methods for accounting for censoring and competing risk of death. We have clarified the purpose of using IPCW with more relevant references as follows:  “First, to additionally account for censoring and competing risk of death that differs between the study and treatment group, we included inverse probability of censoring weighting (IPCW), estimated using a logistic regression with the censoring due to death as the dependent variable and baseline covariates as independent variables[33, 34], along with IPTW in analytical outcome models…” **(Method, Statistical Analysis, Page 14, Lines 270-274)** |
| 13. I am concerned the clinical significance of the outcomes is not sufficiently considered (separately to the statistical significance), which is a risk with large sample sizes like in this research. For example, the primary result "the study vs. comparison group had higher risk of worsening pain (aRR, 1.04 [95% CI, 1.02-1.06]; P < .001)…" is undoubtedly statistically significant but is a 4% difference between groups of clinical importance? Also, when comparing this study and the authors' earlier study it is suggested "…the magnitude of these associations differs, with a smaller effect on worsening pain (RR=1.04 vs 1.03)…"; here I would question a 4% worsening of pain is "different in magnitude" to a 3% worsening of pain. Suggest discussing clinical significance ideally referencing reasonable minimum differences established clinically, and perhaps moderating language in some cases. | We agree with the review on this comment and appreciate the reviewer’s suggestion in framing clinical significance by referencing reasonable minimum clinically important differences (MCIDs). However, the lack of consensus in MCIDs for our studied the clinical and adverse outcomes prevented us from drawing a valid clinical inference.  Alternatively, per our reviewers’ suggestion, we addressed the clinical importance of our estimates by additionally calculating (1) the adjusted absolute risk per group and (2) adjusted risk differences (RDs) between groups for each outcome of interest, including worsening pain. The absolute estimates are now added along with the relative estimates in **Tables 2 and 3**. We described this in Methods as follows:  “We also calculated the adjusted absolute risk of the study and treatment group and the adjusted risk difference (RD) between groups for each clinical and ORAE outcome using a SAS macro [33].” **(Method, Statistical Analysis, Page 14, Lines 267-269)**  We also provided the RD estimates in the Abstract and Results as follows:  **Abstract**: “was associated with a higher risk of worsening pain (relative risk:1.04 [95% CI, 1.02, 1.06]; P=< .001; risk difference (RD): 1.1% [95% CI, 0.6%, 1.6%]) and a higher incidence rate of pain-related hospitalizations (incidence rate ratio [IRR]:1.13 [95% CI, 1.05, 1.22]; P=.003; RD: 1.21 [95% CI, 0.39, 1.89] per 1000 patient-years) and pain-related ED visits (IRR=1.17[95% CI, 1.07, 1.29]; P= .003; RD: 0.85 [95% CI, 0.29, 1.41] per 1000 patient-years), with no difference in physical function, depression, OUD, and OD.” **(Abstract, Pages 3-4, Lines 26-31)**  **Results:**  “After covariate adjustment, the study vs. comparison group had a higher risk of worsening pain (aRR, 1.04 [95% CI, 1.02, 1.06]; *P* < .001) with an absolute increase in the risk of worsening pain by 1.1% (95% CI, 0.6%, 1,6%) during the 1-year follow-up.” **(Results, Page 16, Lines 312-315)**  “After covariate adjustment, the study vs. comparison group had higher incidence rates of pain-related hospitalizations (aIRR, 1.12 [95% CI, 1.04,1.21]; *P* = .004) and pain-related ED visits (aIRR, 1.17 [95% CI, 1.05, 1.30]; *P* = .004) during follow-up. The adjusted absolute increase in the incidence of pain-related hospitalizations and ED visits in the study vs comparison group were 1.12 cases per 1000 patient-years (95% CI, 0.39, 1,89) and 0.85 cases per 1000 patient-years (95% CI, 0.29, 1.41).” **(Results, Page 16, Lines 323-329)**  We would also like to clarify a typo regarding the magnitude of worsening pain in our current vs previous study [8]. The relative risk (RR) should be 1.04 vs 1.13, rather than 1.04 vs 1.03. We have corrected this RR and added RD estimates to facilitate clinical interpretation as follows”  “the magnitude of these associations appear to differ, with smaller relative and absolute effects on worsening pain (RR=1.04 vs 1.13; RD= 1.1% vs 3.9%), pain-related hospitalization (IRR=1.13 vs 1.37; RD=1.12 vs 3.96 per 1000 patient-years), and pain-related ED visit (IRR=1.17 vs 1.49; RD=0.85 vs 3.02 per 1000 patient-years) associated with the opioid-triggered interactions vs antidepressant-triggered interactions [8]. **(Discussion, Page 18, Lines 372-376)**  In the **Discussion and conclusions**, we have softened our conclusions and clinical suggestions given the small-to-moderate relative and absolute estimates observed in our study sample as follows:  “We observed small magnitude of RR (4%) and RD (1.1%) for worsening pain and moderate magnitude for pain-related hospitalizations (RR=13%; RD=1.21 per 1000 patient-years) and ED visits (RR=17%; RD=0.85 per 1000 patient-years). These findings suggest that initiating CYP2D6-metabolized opioids on existing antidepressants that strongly or moderately inhibit the CYP2D6 enzyme was associated with worsening pain control and increased risk for pain-related medical encounters, although their RRs and RDs appear to be small to moderate during the follow-up period (up to 1 year) among older NH residents.” **(Discussion, Pages 17-18, Lines 354-361)**  “The present study provides referential data for clinicians regarding the safety of initiation of CYP2D6-metabolized opioids on existing antidepressants (i.e., opioid-triggered interaction), which was predominantly used by older NH residents for treating their comorbid depression and chronic pain. Among patients whose opioid-antidepressant interaction was triggered by the opioids, concomitant use of CYP2D6-metabolized opioids with CYP2D6-inhibiting (vs CYPD2D6-neutral) antidepressants was associated with an increased risk of worsening pain and risk of pain-related hospitalizations and ED visits. However, the relative and absolute risks of these outcomes associated with opioid-triggered interactions appear to be low to moderate, compared to the estimates associated with antidepressant-triggered interactions [8]. Overall, our study along with the prior study[8] suggests that when co-use of CYP2D6-metabolized opioids and antidepressants is clinically needed, selection of CYP2D6-neutral antidepressants, rather than CYP2D6-inhibiting antidepressants, may reduce the risk of worsening pain and pain-related hospital and ED visits. **(Discussion, Pages 19-20, Lines 399-411)**  Conclusions: “In this cohort study of older NH residents who received antidepressants, concomitant use of CYP2D6-inhibiting (vs. CYP-neutral) antidepressants with newly-prescribed CYP2D6-metabolized opioids was associated with increased risk of worsening pain and hospital and ED visits due to pain, although the relative and absolute risks are small to moderate. No association was found for physical function, depressive symptoms, OUD, and OD. The findings, consistent with a prior study [8], suggested that clinicians should be aware of potential worsening pain and hospital and ED visits due to pain among patients who used CYP2D6-metabolizing opioids concomitantly with antidepressants, particularly those with CYP2D6-inhibiting antidepressants.” **(Discussion, Page 21, Lines 441-449)** |
| 14. [MINOR] "Our observed null association with OD is also supported by pharmacokinetic…" Be careful with wording here - absence of evidence is not evidence of absence. | We have revised the sentence as follows:  “No difference in OD was observed between the study and comparison group, which is consistent with preclinical [37, 38] and pharmacokinetic modeling studies [39, 40], suggesting that use of CYP2D6 inhibitors alone with oxycodone is not associated with increased oxycodone and oxymorphone concentration, a major underlying cause of OD. **(Discussion, Page 19, Lines 394-398)** |
| 15. [MINOR] Considered together, the main publication and supplementary materials report a very large number of p-values with the attendant risk of Type I error(s). I appreciate they are included for completeness however I would encourage consideration of omitting some - particularly in the supplementary materials, noting 95% CIs suffice in many cases | We thank the reviewer for this comment. However, per PLOS editorial request, all results are expressed with 95% CIs as well as p values. |
| 16. Given the complexity of the data management and statistical analyses, I would strongly encourage the authors to include their code to facilitate understanding and replicability. At a minimum this would include their statistical modelling code and, ideally, code developed to produce variables of interest including outcomes, exposures, and other covariates. | We have provided examples of SAS codes of statistical modeling for our clinical and adverse outcomes in the pre-specified protocol (**S1 Text**). |
| **Reviewer 2:** |  |
| 1. Introduction: While the authors discuss the potential mechanisms underlying interactions between these drug groups and their impact on outcomes to some extent, further elaboration on these mechanisms would strengthen the study's background and justification. This would help establish clearer hypotheses for the three outcomes: worsening depressive symptoms, impaired physical functioning, and increased pain intensity. | We have provided additional information on opioid-related adverse events in the Introduction as follows:  “The reduction in analgesic effects associated with opioid-antidepressant interaction results in worsening of pain [4-8], and persistent pain can lead to increased risk for impaired physical functioning and mental health problems, including depression [9]. The reduction in analgesic effects can also result in opioid-related adverse events (ORAEs), including pain-related hospitalizations and emergency department [ED] visits) [4, 8, 10]. **(Introduction, Page 7, Lines 79-84)** |
| 2. Methods - Covariates: Why was the calendar year not included as a covariate? Prescribing patterns for opioids and antidepressants, as well as clinical guidelines and care facility utilization, likely changed over time and could influence the outcomes. | We have accounted for the calendar year in which eligible residents entered the cohort, described as follows:  “We also accounted for ..., and year of cohort entry.” **(Method, Page 13, Line 237)**  We presented the results in **Table 1 and S4 Table**. Overall, the results are consistent with what we reported previously. |
| 3. Sensitivity Analyses: It would be valuable to perform an additional analysis restricting antidepressant users to those with a treatment indication of depression. Since antidepressants in older adults may also be prescribed for non-depressive indications (e.g., pain), excluding individuals with such indications could help clarify and rule out the possibility that worsening pain intensity was due to selection bias—specifically, the inclusion of individuals already using antidepressants for pain who may have experienced greater worsening in pain intensity. | We would like to clarify that in sample selection, we did restrict our sample to those with diagnoses that are FDA-approved or off-label use for antidepressants as follows:  “To minimize confounding by disease conditions, we restricted the study sample to those with a diagnosis of chronic pain for opioid therapy initiation within 1 month and conditions that are FDA-approved or off-label use for antidepressants within 6 months before the cohort entry.” **(Method, Study Sample, Page 9, Lines 137-140)**  We refrained from restricting to residents with a diagnosis of depression only because antidepressants can be used for treating neuropsychiatric symptoms, including not only depression but also other symptoms, such as anxiety. These symptoms are prevalent in nursing home residents.  Because some antidepressants (i.e., SNRI and TCAs) have additional analgesic effects and thus are commonly used for treating pain, we did a sensitivity analysis as follows:  “Second, to understand whether associations varied by antidepressant classes according to whether they had additional analgesic effects (i.e., serotonin and norepinephrine reuptake inhibitors [SNRIs] and tricyclic antidepressants) or not (SSRIs and other antidepressants), we stratified analysis for these two groups”. **(Method, Statistical Analysis, Page 14, Lines 275-278)**  The results are shown in **Table 4** and reported in Results. Overall, we observed results consistent with the main analysis for all clinical outcomes, OUD, and OD adverse outcomes in antidepressant users with or without additional analgesic effects. Association for pain-related hospitalizations and ED visits were only significant in antidepressant users without additional analgesic effects, not those with additional analgesic effects likely due to a small sample size. |
| 4. If data on treatment indications for antidepressants and opioids are unavailable, this limitation should be acknowledged, as confounding by indication is a significant concern in observational research. | Medicare Part D prescription event data do not provide information on the disease indication(s) for which a drug is prescribed, which we have acknowledged as follows:  “Medicare Part D data provided no information on disease indications for which a drug is prescribed. To address this issue, we required eligible residents to have a diagnosis of chronic pain (for opioid initiation) and a diagnosis of indications FDA-approved or off-label use (for antidepressant treatment) during the baseline period to minimize potential confounding by indication.” **(Discussion, Limitations, Page 20, Lines 425-429)** |
| 5. Sensitivity Analyses - Clarification: The sensitivity analyses section would benefit from further explanation of the purpose of each sensitivity analysis and what potential biases or issues they aim to address. This may not be immediately clear to all readers. | We clarified the purpose of each proposed sensitivity analysis as follows:  “First, to additionally account for censoring and competing risk of death that differs between the study and treatment group, we included inverse probability of censoring weighting (IPCW), estimated using logistic regression with the censoring due to death as the dependent variable and baseline covariates as independent variables[34, 35], along with IPTW in analytical outcome models. Second, to understand whether associations varied by antidepressant classes according to whether they had additional analgesic effects (i.e., serotonin and norepinephrine reuptake inhibitors [SNRIs] and tricyclic antidepressants) or not (SSRIs and other antidepressants), we stratified analysis for these two groups. Third, to understand whether associations varied by CYP2D6-metabolized opioids, we stratified by specific opioid drugs. Fourth, to minimize potential competing effects arising from use of other CYP2D6 inhibitors, we restricted the analysis to patients without use of other CYP2D6-inhibiting medications at baseline. Fifth, to address the potential non-independence within individual observations due to cohort re-entry, we included the first eligible observation of each study sample.” **(Method, Pages 14-15, Lines 270-284)** |
| 6. Discussion: In the first paragraph of the discussion section, the authors state that "CYP2D6 enzyme led to worsening pain control." The phrase "led to" implies causality, which is inappropriate in this context. The statement should be revised to avoid suggesting causation | We agree with the reviewer and have revised the language as follows:  “CYP2D6-metabolized opioids …was associated with worsening pain control” **(Discussion, Page 18, Line 358)** |
| 7. Public Health Impact: To enhance the discussion of public health implications, the authors could calculate an attributable fraction for the population. This would estimate how many excess cases of hospitalizations or ED visits could be prevented by prescribing CYP2D6-neutral antidepressants alongside CYP2D6-metabolized opioids. | We agree with the reviewer on this comment. To enhance the interpretation of the clinical relevance of our estimates, we have now additionally calculated: 1) adjusted absolute risk for the study and treatment group; and (2) risk difference (RD) between the study and treatment group for each outcome of interest in **Table 2 and 3**.  We refrained from providing the number needed to treat (NNT) or harm (NNH) per prior literature [PMID: 19438480] because of the potential for misinterpretation. For example, an NNT of 7 was often incorrectly interpreted as “seven patients would have had to be treated for three years to prevent event” without considering its probability. In our study, because an NNT or NNH number is calculated as an inverse of a difference in incidence rate per 1000 patient-years, the interpretation needs to be expressed in terms of 1000 patient-years, which may not be easy to understand and interpret in clinical settings.  We have included these numbers in the Abstract, Results, and Discussion. We have also revised our conclusions based on the relative risk and risk differences for our outcomes as follows:  “In this cohort study of older NH residents who received antidepressants, concomitant use of CYP2D6-inhibiting (vs. CYP-neutral) antidepressants with newly-prescribed CYP2D6-metabolized opioids was associated with increased risk of worsening pain and hospital and ED visits due to pain, although the relative and absolute risks are small to moderate. No association was found for physical function, depressive symptoms, OUD, and OD. The findings, consistent with a prior study [8], suggested that clinicians should be aware of potential worsening pain and hospital and ED visits due to pain among patients who used CYP2D6-metabolizing opioids concomitantly with antidepressants, particularly those with CYP2D6-inhibiting antidepressants. ” **(Discussion, Page 21, Lines 441-449)** |
| 8. Limitations: Regarding the first limitation, how large is the group of individuals whose prescriptions were self-paid or covered by non-Medicare programs? Including this information would provide additional context | Per suggestion, we have added the descriptions as follows:  “For example, 6% of Medicare beneficiaries were veterans in 2022[41] and may obtain their prescriptions through veterans’ insurance program[42].” **(Discussion, Limitations, Page 20, Lines 417-419)** |
| 9. Prescriber Information: Is there any data on prescriber qualifications (e.g., general practitioners, psychiatrists, or other specialists)? If prescriber characteristics might influence drug selection and treatment outcomes, this limitation should be discussed. | We reframed from including prescribers because types of prescribers are not associated with resident outcomes and thus are not potential confounders. In propensity score modeling, only true variables (both associated with drug exposure and outcomes) or potential confounders (associated with outcomes only) are recommended to be considered [PMID: [21818162](https://pubmed.ncbi.nlm.nih.gov/21818162/);[16624967](https://pubmed.ncbi.nlm.nih.gov/16624967/)] |
| 10. Tables: The presentation of findings in Tables 2 and 3 could be improved by using forest plots, which would make the results more visually engaging and easier to interpret compared to the current monotone tabular format. | We appreciate the reviewer for this suggestion. In this revision, we provided additional estimates (absolute risk per group and risk difference between groups) for each outcome of interest, along with the crude estimates and relative estimates. The current tabular format allows including all estimates both in relative and absolute forms, sufficiently presenting our key results, compared to forest plots. |
| **Reviewer 3:** |  |
| 1. First, I am concerned that the CYP2D6 inhibiting antidepressants were not classified appropriately. For example, some of the medications listed in the CYP2D6 neutral group actually have CYP2D6 inhibiting properties, albeit weaker than paxil and prozac (i.e., sertraline, desvenlafaxine, citalopram). If duloxetine (a mild to moderate 2D6 inhibitor) was grouped with paxil and prozac, shouldn't you group other mild or moderate 2D6 inhibitors? | We classified antidepressants according to whether they have strong/ moderate inhibition or mild/ no inhibition of the CYP2D6 enzyme based on multiple sources, including FDA, Micromedex, Lexicomp, and Drug Interaction databases.  We have clarified our sources in the Methods as follows:  “The classification of antidepressants was made based on multiple sources of drug interactions, including the US Food and Drug Administration [16], Micromedex, Lexicomp, and Drug Interaction Database (DIDB®) [17].” **(Methods, Key Exposure, Page 10, Lines 168-171)**  While sertraline can inhibit the CYP2D6 enzyme, its inhibition is weak and thus likely to have little or no impact on the metabolism of the enzyme. Thus, we classify sertraline and other drugs with weak inhibition of CYP2D6 enzyme in a group. Per our databases including FDA, duloxetine is classified as a moderate CYP2D6 inhibitor. |
| 2. There is no doubt that 2D6 metabolism is impacted by various antidepressants. Whether this is a clinically meaningful impact is a whole different story. In short, I am not convinced the authors established the theoretical foundation for focusing on 2D6.   Some recent studies have suggested that 2D6 inhibition itself is inadequate to meaningfully interfere with oxycodone metabolism. See PMID 20857093 for more details: "paroxetine pretreatment inhibits cyp2d6 without inducing relevant changes in oxycodone exposure and partially blunts the pharmacodynamic effects of oxycodone due to intrinsic pharmacological activities." Also see PMID 21142269 "the effect of paroxetine on plasma concentrations of oxycodone was negligible."  Overall, I am not convinced that the association observed here is even driven by CYP2D6 effects. Namely, several of the antidepressants in the 2D6 group are well known to be anticholinergic and even land on the Beers list of geriatric medications. How do we know it's the 2D6 that's the underlying cause? | We appreciate the different views brought up by the reviewer. However, as described in the Introduction, we relied on the underlying pharmacokinetic (PK) feature of these 4 opioids—codeine, hydrocodone, oxycodone, and tramadol, all of which are required metabolism by the CYP2D6 enzyme to their potent analgesic metabolites, which have been well-established. Our study examined a total of 7 clinical and adverse outcomes due to inadequate analgesia associated with opioid-antidepressant PK interaction.  In Beers Criteria, opioids are included along with other central nervous system (CNS) drugs because of pharmacodynamic (PD) CNS effects, which can lead to the risk of falls/fractures.  The reviewer mentioned 2 preclinical studies [PMID 20857093; 21142269] that focused on oxycodone among 12 healthy participants, suggesting that use of CYP2D6 inhibitors (e.g., paroxetine) with oxycodone doesn’t seem to increase oxycodone concentration, which is a key underlying cause of opioid overdose (OD). We have included this in the Discussion as follows:  “No difference in OD was observed between the study and comparison group, which is consistent with preclinical [37, 38] and pharmacokinetic modeling studies [39, 40], suggesting that use of CYP2D6 inhibitors alone with oxycodone is not associated with increased oxycodone and oxymorphone concentration, a major underlying cause of OD.” **(Discussion, Page 19, Lines 394-398)** |
| 3. In clinical practice, cymbalta and paxil also tend not to be 1st line meds for bread and butter depression. This raises questions about unmeasured confounding. Could it just be that the people receiving paxil and cymbalta are sicker at baseline -- and have more comorbidities -- than the other group, contributing to the observed association?   I appreciate the use of IPTWs w/ Standard diffs showing good balance between the groups. However, I am still concerned about residual confounding (i.e., there was no adjustment for the amount of time people were on opioids, the dosage of opioids, the type of opioids, etc.) This could very well confound the observed association. The fact that the study group had 61.6% using an adjuvant analgesic vs 37% in the control group also makes me wonder if the study group just had more baseline pain levels and would have had higher pain related healthcare encounters no matter what antidepressant. | We appreciate the reviewer for this comment. We have tried our best effort to measure potential confounders from both Medicare claims data as well as the Minimum data set (MDS) 3.0 assessment. As shown in Table 1, a total of 31 potential confounders, including comorbidities, use of medications other than opioids, pain intensity, etc. have been accounted for and are balanced between the study and treatment group.  No adjustment for the duration, dosage, and types of opioids at baseline because everyone included in our study was required to initiate opioids on existing antidepressants, without any such prescription filled at the baseline (6 months) before the cohort entry (i.e., the day 1 of initiation of the opioids with antidepressants). Also, we observed little variation in daily dosage of the CYP2D6-metabolized opioids that were initiated on the cohort entry, with 98% of residents receiving < 5 morphine milligram equivalents per day, which is the main reason we refrained from including opioid dosage.  We do agree that there are potential unmeasured confounders that may be in place and have acknowledged this in limitation as follows:  “Sixth, while we accounted for many potential confounders, our results remain subject to unknown or unmeasured confounding.” **(Discussion, Limitations, Page 21, Lines 433-434)** |
| 4. The results don't appear very robust or clinically meaningful enough to justify the authors' conclusions: For example, an 15% vs 11% difference in pain related hospitalization, with a confidence interval of 1.04-1.21, is a very small difference. Should a clinician switch someone who has been stable on prozac to something else when the difference is merely 15% vs 11% (w/ a 95%CI abutting 1.00)? Several of the secondary analyses seemed to have no association between 2D6 inhibitors vs 2D6 neutral meds and the outcome variables of interest. | We would like to clarify that we calculated the incidence rate of opioid-related adverse outcomes, including pain-related hospitalizations, and the rate is expressed as the number of events per 1000 patient-years, rather than %.  We observed that the crude rate of pain-related hospitalization is 15 vs 11 per 1000 person-years for the study vs comparison group before the IPTW adjustment (Table 2). After the IPTW adjustment, the adjusted relative rate ratio is 1.12 with a 95% CI of 1.04-1.21, meaning that the adjusted incidence rate is 12% higher in the study vs comparison group.  To facilitate the clinical interpretation, in this revision, we have provided additional estimates of 1) absolute risk per group and 2) risk difference between groups for each outcome in **Tables 2 and 3**. We have included these numbers in the Abstract, Results, and Discussion.  We have also revised our conclusions based on the relative risk and risk differences for our outcomes as follows:  “In this cohort study of older NH residents who received antidepressants, concomitant use of CYP2D6-inhibiting (vs. CYP-neutral) antidepressants with newly-prescribed CYP2D6-metabolized opioids was associated with increased risk of worsening pain and hospital and ED visits due to pain, although the relative and absolute risks are small to moderate. No association was found for physical function, depressive symptoms, OUD, and OD. The findings, consistent with a prior study [8], suggested that clinicians should be aware of potential worsening pain and hospital and ED visits due to pain among patients who concomitantly used CYP2D6-metabolizing opioids and antidepressants, particularly those with CYP2D6-inhibiting antidepressants.” **(Discussion, Page 21, Lines 441-449)**  Most of the sensitivity data show results consistent with the main studies. When stratifying by specific opioids, we observed associations for pain-related hospitalizations and ED visits are not significant for codeine, largely due to its small sample within the stratified group. |
| 5. I'm a little confused why you required residents to have coma or severe cognitive impairment - this could confound the pain assessment and the reasons for hospitalizations. Pain assessment would have much higher variability for a person with cognitive impairment. If it's being assessed by different providers, there would also be more operator variability. This can greatly skew your results. | We apologize for the error. There should be no coma or severe cognitive impairment. We have now modified the phrase as follows:  “6) no coma or severe cognitive impairment…” **(Methods, Study Sample, Page 10, Line 151)** |
| 6. Pain related hospitalization and ED visits were defined as encounters with a primary or secondary diagnosis of a chronic pain condition: there is a lot of room for error here. Lots of chronic pain is coded as a hospital problem, even if the plan is to just continue home medications. It does not necessarily mean that the healthcare encounter was at all a result of the pain. There's no way to know that the pain was current and pertinent to the admission or ED visit. | We have acknowledged this as a limitation as follows:  “Fourth, because we relied on resident reports for clinical outcomes and administrative claims data for pain-related hospitalizations and ED visits, measurement bias was possible, although such bias is likely nondifferential between the study and comparison groups.” **(Discussion, Limitations, Pages 20-21, Lines 4829-431)** |
| 7. On page 9, you say we classified residents with score changes equal to 0 that remained moderate to severe as having worsening outcomes. Similarly, you classified residents with score change of 0 that had no-to-mild pain as improving outcome. Those two scenarios are by definition the same outcome, not worsening or improving outcomes? | We have further clarified this as follows:  “The continuous score changes of these symptoms included scores >0 (worsening), <0 (improving), and 0 (no change). Patients with no change in symptoms include two clinically different patient subgroups—(1) patients who remained no-to-mild from baseline to follow-up, suggesting their symptoms were controlled; and (2) patients who remained moderate-to-severe from baseline to follow-up, suggesting their symptoms were uncontrolled or got worse. Thus, similar to previous studies [6, 16], we classified residents with score changes >0 or equal to 0 that remained moderate to severe as having worsening outcomes, and otherwise as improving outcomes (score changes <0 or score changes equal to 0 that remained no-to-mild symptoms).” **(Methods, Page 12, Lines 206-214)** |
| 8. On page 6, you say among older NH patients who concomitantly use both CYP2D6-metabolized opioids and antidepressants, 80% are those who received antidepressants first followed by opioids. What is the source of this statistic? | We published a paper focusing on concomitant opioid-antidepressant use among the nursing home population [reference 8]. Using the 100% nursing home sample, we found that 20% of opioid-antidepressant concomitant users were those who used CYP2D6-metabolized opioids followed by initiation of antidepressants (i.e., antidepressant-triggered interaction) [8], whereas 80% were those who used antidepressants followed by initiation of CYP2D6-metabolized opioids followed by initiation of (i.e., opioid-triggered interaction). We have clarified as follows:  “however, these patients constitute only 20% of older NH patients who concomitantly used both CYP2D6-metabolized opioids and antidepressants according to our preliminary data using the 100% NH sample [8]. The predominant (80%) concomitant users of both drugs in NHs …” **(Introduction, Page 8, Lines 100-103)** |
| **Reviewer #4** |  |
| 1. The methods are generally well chosen considering interdependency of individuals included several times and repeated outcome measurements and covariate balance and adjustment. However, re-entering of previous users may dilute the effect due to depletion of susceptibles, even though the influence of drug-drug interactions may be virgin. However due to the complexity of IA depletion of susceptibles may still be relevant? Please comment on this potential bias. | By definition, depletion of susceptible subjects occurs when patients who remain on drugs are those who can tolerate and have a low chance of developing drug adverse events, whereas patients who are high-risk and susceptible to the adverse effects are depleted from the populations. The reviewer raised a concern on the potential depletion of susceptible patients in our study sample given that individuals can re-enter the cohort when meeting all eligibility criteria. Per the definition, the reviewer’s concern will be valid only when patients who re-entered the cohort had better tolerance and thus had a ow chance of developing adverse events due to the opioid-antidepressant interaction in the early observed episodes. For patients who entered the cohort once without re-entering, that is because they are high-risk and have a high chance of experiencing drug adverse events in the first observed episode. To validate the reviewer’s concern, we restricted our sample (n=127,200) to their first observed episode and compared the crude incident rate of opioid-related adverse event (ORAE) outcomes among patients who later re-entered the cohort (n=9860) vs those who never re-entered (n=117,340). We showed the results in the following table. \| **Crude incidence rate per 1000 PY (event/PY)** \| \| **P-value^1^**; ORAE outcomes \| Patients who later re-entered (n=9860) \| Patients who did not re-enter (n=117,340) \|; Pain-related hosp. \| 11.63 (97/8338) \| 11.72 (1092/93140) \| 0.94; Pain-related ED \| 6.34 (53.8338) \| 6.51 (606/93140) \| 0.87; OUD^2^ \| 7.09 (59/8326) \| 5.73 (533/93024) \| 0.12; OD^2^ \| 4.08 (34/8326) \| 4.20 (391/93024) \| 0.87 ^1^ was generated using a Poisson or negative binomial model ^2^ restricted to samples without OUD or OD during the 6-month baseline. As shown, the incidence rates of all ORAE outcomes did not statistically differ between groups, which did not support the concern on depletion of susceptible patients—that is, patients who later re-entered the cohort are those who had a low chance of developing adverse events and patients who entered the cohort only for one time are those who are high-risk, experiencing outcomes in earlier episodes. In conclusion, there is no evidence supporting the depletion of susceptible patients in our sample selection during which individuals are allowed to re-enter the cohort, and thus there is no bias relevant to that concern. We also conducted a sensitivity by restricting the first observation of each study sample (**Methods, Page 15, Lines 281-282**), and the results of this sensitivity (shown in **S9 Table**) were consistent with that from the main analysis, suggesting the allowing cohort re-entry doesn’t dilute our estimates, |
| 2. According to the authors one day of overlap was sufficient to qualify for concurrent use. The authors allowed gaps of less than 15 days between prescriptions. Please detail how episodes of concurrent exposure were constructed and how this may effect the risk estimates during the one year of follow-up. This is important to be reported to enable reproduceability of results. | We have elaborated on the details of how episodes of concurrent use were constructed as follows:  “For each drug, we determined days of drug use according to days’ supply and dispending date of prescriptions. We then constructed episodes of concomitant opioid-antidepressant use by identifying the continuous number of overlapping days on which a CYP2D6-metabolized opioid was used along with an antidepressant drug. When constructing episodes of concomitant opioid-antidepressant use, we allowed a gap of fewer than 15 days to account for delays in fills of prescription drugs of interest.” (**Methods, Page 10-11, Lines 171-176)**  Noted that only episodes in which opioids were initiated on existing antidepressants are eligible in our analysis for measuring the risk of outcomes of interest, which was delineated in our study sample selection. Risk of outcomes was captured in the follow-up, which starts from the index date (i.e., the initiation of opioids on the existing antidepressant use) and ends at one of the censoring points (death, NH discharge, study end [12/31/2025], and end of 1-year follow-up). We used intention-to-treat analysis (ITT) without censoring individuals when they stopped CYP2D6-metabolized opioids or antidepressants or switched from CYP2D6-inhibiting to CYP2D6-neutral antidepressants (or vice versa). The ITT tends to provide conservative estimates, which have acknowledged this in the limitation as follows:  “Seventh, we used intention-to-treat analysis without censoring patients when they stopped CYP2D6-metabolized opioids or antidepressants or when they switched from CYP2D6-inhibiting to CYP2D6-neutral antidepressants (or vice versa) during the follow-up, which may yield conservative effect estimates, compared to per protocol analysis.” **(Discussion, Limitations, Page 21, Lines 434-438)** |
| 3. Would any information of the dosage of drugs be available for the analysis and how could this consideration have affected the findings? | Medicare Part D data don’t have information on drug dosage, that is how much medication the doctor wants a patient to take at each time (e.g., 10 mg twice per day) for each prescription. It only provides the drug strength of a drug product (5 mg, 10mg, or 2 mg), which indicates the amount of active ingredient in each drug.  We refrained from accounting for drug strength because of little variation in the strength of antidepressants used in the nursing home sample. We attempted to calculate daily morphine milligram equivalents (MME) of the initiated opioid using an equation: [drug strength/ days supple] X conversion factor. We also observed little variation in daily MME of the initiated CYP2D6-metabolized opioids, with 98% of residents receiving < 5 MME per day. |
| 4. The authors consider concomitant use of other CYP inhibitors and inducers at baseline and by exclusion in sensitivity analyses. a) For reproducabiliyty reasons, I could not find any information of how these were identified and which drugs fell into which category. b) Moreover, it would have been interesting to explore the association between the burden of inhibitors and inducers on the outcomes during follow-up in a time varying manner, but also depending on how concurrent use episodes of the opioid drugs of interest were constructed. It is not clear why the other drugs metabolized by CYP would have more or less impact than the antidepressants under investigation, in particular, if used in a regular manner. Please comment on that. | For 4. a, we have included the references as follows:  “Medication use included use of other CNS medications, use of other CYP2D6 inhibitors[2, 16, 25], use of CYP3A4 inhibitors[2, 16, 25], use of CYP2D6 inducers[2, 16, 25],” **(Methods, Pages 12-13, Lines 225-226)**  For 4.b, we would like to clarify that for opioid-related adverse outcomes, we captured the total counts of events during the entire follow-up (up to 1 year), making it challenging to incorporate any covariates, such as the burden of inhibitors and inducers as time-varying factors in the analysis. Also, there is a small proportion of our sample who had use of other CYP2D6 inhibitors (0.8%) and CYP2D6 inducers (3.6%), suggesting that the use of these medications may not have a substantial impact on our estimates.  For 4.c, we have justified why we accounted for these drugs as follows:  “We accounted for use of other CYP2D6 inhibitors because they may compete with CYP2D6-inhibiting antidepressants for the CYP2D6 enzyme [25], use of CYP3A4 inhibitors because the studied opioids are also metabolized at least to some extent via the CYP3A4 enzyme, which could also affect pain-related outcomes[2, 26], and use of CYP2D6 inducers because they increase the metabolic activity of the CYP2D6 enzyme, which can also result in inadequate analgesia and affect pain-related outcomes[2, 26].” **(Methods, Page 13, Lines 227-232)** |
| 5. Communication of absolute risk differences and number needed to harm could be clinically relevant putting the reported 4% higher risk of worsening pain and 13% and 17% higher incidence rates of pain-related hospitalizations and pain-related ED visits into perspective. | We have provided (1) adjusted absolute risk per group and (2) risk difference between groups for all clinical and opioid-related adverse outcomes. These two absolute estimates are considered sufficient and preferable to the number needed to treat (NNT) or harm (NNH) per prior literature [PMID: 19438480].  We refrained from providing NNT or NNH because of the potential for misinterpretation. For example, an NNT of 7 was often incorrectly interpreted as “seven patients would have had to be treated for three years to prevent event” without considering probability. In our study, because an NNT or NNH number is calculated as an inverse of a difference in incidence rate per 1000 patient-years, the interpretation needs to be expressed in terms of 1000 patient-years, which may not be easy to understand and interpret in clinical settings.  We have included estimates both in relative and absolute numbers in the Abstract, Results, and Discussion. We have also revised our conclusions based on the relative risk and risk differences for our outcomes as follows:  “In this cohort study of older NH residents who received antidepressants, concomitant use of CYP2D6-inhibiting (vs. CYP-neutral) antidepressants with newly-prescribed CYP2D6-metabolized opioids was associated with increased risk of worsening pain and hospital and ED visits due to pain, although the relative and absolute risks are small to moderate. No association was found for physical function, depressive symptoms, OUD, and OD. The findings, consistent with a prior study [8], suggested that clinicians should be aware of potential worsening pain and hospital and ED visits due to pain among patients who used CYP2D6-metabolizing opioids concomitantly with antidepressants, particularly those with CYP2D6-inhibiting antidepressants.” **(Discussion, Page 21, Lines 441-449)** |
| **Editorial requests** |  |
| 1. We ask every co-author listed on the manuscript to fill in a contributing author statement, making sure to declare all competing interests. | All the co-authors have declared their competing interests. |
| 2. Please upload any figures associated with your paper as individual TIF or EPS files with 300dpi resolution at resubmission. | All figures are uploaded as an individual TIF with 300 dpi. |
| 3. Please ensure that the paper adheres to the PLOS Data Availability Policy. | We have addressed the PLOS Data Availability Policy. |
| 4. We expect all researchers with submissions to PLOS in which author-generated code underpins the findings in the manuscript to make all author-generated code available without restrictions upon publication of the work. | We have included example SAS codes of the statistical models for the key findings in the pre-specified protocol plan (**S1 text**). |
| FORMATTING - GENERAL |  |
| Abstract: Please structure your abstract using the PLOS Medicine headings (Background, Methods and Findings, Conclusions). Please combine the Methods and Findings sections into one section. | We have structured the Abstract per instruction. |
| At this stage, we ask that you include a short, non-technical Author Summary of your research to make findings accessible to a wide audience that includes both scientists and non-scientists. The Author Summary should immediately follow the Abstract in your revised manuscript. | We have provided the author's summary statement. |
| Please express the main results with 95% CIs as well as p values. When reporting p values please report as p<0.001  and where higher as the exact p-value p=0.002, for example. Throughout, suggest reporting statistical information as  follows to improve clarity for the reader "22% (95% CI [13%,28%]; p</=)". Please be sure to define all numerical values at first use. | We have expressed the main results with 95% CIs as well as p values in the main text per instruction. |
| Please include page numbers and line numbers in the manuscript file. Use continuous line numbers (do not restart the  numbering on each page). | We have included page numbers and link numbers in the manuscript file. |
| Please cite the reference numbers in square brackets. Citations should precede punctuation. | We have cited the reference numbers in square brackets per instruction. |
| FIGURES AND TABLES |  |
| Please provide titles and legends for all figures and tables (including those in Supporting Information files). | We have provided titles and legends for all figures included |
| Please define all abbreviations used in each figure/table (including those in Supporting Information files). | We have defined all abbreviations used in each figure/table. |
| SUPPLEMENTARY MATERIAL |  |
| Please cite your Supporting Information as outlined here:  https://urldefense.com/v3/__https://journals.plos.org/plosmedicine/s/supportinginformation__;!!  KGKeukY!whsXxcky8MVh_TgghR9NO4JS9KHJUoYix1aiJwArdnjwgV80YNXlVhhEm_  qwEFr9fI1mjI1-d71TFJn-7s$ | We have followed the instructions for our supporting information. |
| REFERENCES |  |
| PLOS uses the numbered citation (citation-sequence) method and first six authors, et al. | We have followed the instructions. |
| Please ensure that journal name abbreviations match those found in the National Center for Biotechnology Information (NCBI) databases. | We have followed the instructions. |
| Where website addresses are cited, please include the complete URL and specify the date of access (e.g. [accessed:  12/06/2023]). | We have followed the instructions. |
| OBSERVATIONAL STUDIES |  |
| Abstract: Please include the study design, population and setting, number of participants, years during which the study took place (enrollment and follow up), length of follow up, and main outcome measures. | We have followed the instructions. |
| Please ensure that the study is reported according to the STROBE (or appropriate STOBE extension) guideline. | We have included the STROBE guideline for our study as follows:  “This study followed the STROBE reporting guideline (**S1 STROBE Checklist)**. Data analyses were performed as per a pre-specified protocol between January and December 2024 (**S1 Text**).” |
| For all observational studies, in the manuscript text, please indicate: (1) the specific hypotheses you intended to test,(2) the analytical methods by which you planned to test them, (3) the analyses you actually performed, and (4) when reported analyses differ from those that were planned, transparent explanations for differences that affect the reliability of the study's results. If a reported analysis was performed based on an interesting but unanticipated pattern in the data, please be clear that the analysis was data driven. | We have followed the instructions. |
| Please state in the Methods section whether the study had a prospective protocol or analysis plan. | We have added a prospective analysis plan in the supplement and described it in the main text as follows:  “This study followed the STROBE reporting guideline (**S1 STROBE Checklist)**. Data analyses were performed as per a pre-specified protocol between January and December 2024 (**S1 Text**).” |
