# Supplementary material for: Association of initiating CYP2D6-metabolized opioids with risks of adverse outcomes in older adults receiving antidepressants: A retrospective cohort study
Source: PLoS Med. 2025 Jun 2;22(6):e1004620. doi: 10.1371/journal.pmed.1004620 (PMC12129234; doi:10.1371/journal.pmed.1004620)
Supplement: Attachment — Submitted filename: Reviewers comments 2nd round.docx [file pmed.1004620.s015.docx]

Dear Authors,

Thank you for your thorough responses and the revisions made to the manuscript. I appreciate your efforts to address my concerns, particularly regarding the inclusion of adjusted absolute risks and risk differences, as well as the clarification on the limitations of the data.

While I still have some residual concerns regarding potential confounding by indication and the lack of study-specific data for certain limitations, I recognize that you have made a genuine effort to address these points. I acknowledge the inherent limitations of observational studies and believe that the remaining aspects are unlikely to significantly alter the overall findings.

Therefore, I strongly recommend that you:

Emphasize the need for caution when interpreting the results in light of the potential confounding by indication, particularly by adding a statement to the conclusion section that acknowledges this potential bias.

I believe that a thorough and transparent discussion of these limitations is crucial for the accurate interpretation of your findings. I recommend acceptance of the manuscript, with these minor revisions.
