# Supplementary material for: Association of initiating CYP2D6-metabolized opioids with risks of adverse outcomes in older adults receiving antidepressants: A retrospective cohort study
Source: PLoS Med. 2025 Jun 2;22(6):e1004620. doi: 10.1371/journal.pmed.1004620 (PMC12129234; doi:10.1371/journal.pmed.1004620)
Supplement: Attachment — Submitted filename: PlosMed Response_4.25.2025.docx [file pmed.1004620.s016.docx]

**Response to Editor’s and Reviewer’s comments on Manuscript ID: PMEDICINE-D-24-03810R2**

| **Reviewer’s Comments** | **Our Response** |
| --- | --- |
| **Reviewer #1** |  |
| Thanks to the authors for their thoughtful and comprehensive responses to my review comments. I am satisfied the authors have addressed all my concerns and I am happy to recommend acceptance.  I also wanted to include a few additional thoughts for the authors' future consideration. I don't think these are necessary for this particular paper because, fortuitously, there were small numbers of patients re-entering the cohort and limited clustering effects. That said, it would be remiss of me to not to be clear on these points for future cases when there is a pronounced lack of independence within the data.  For items 5 and 6 the authors made use of PROC GLIMMIX to fit models accounting for non-independence within the data. The authors then experienced issues with convergence - a problem I have regularly encountered when fitting GLMMs to large datasets. I draw the authors' attention to a SAS paper I have found useful https://urldefense.com/v3/__https://support.sas.com/resources/papers/proceedings18/2179-2018.pdf__;!!KGKeukY!xhyk-whL1jmWXuZyqxtlBEqVv7fDIBEAQmUJ0UbBo8A3o_fvwoLyLuK-OlCLoTVu-l1k4N2Gb9TKcx2wxek$) and a section of the SAS documentation that is important and not at all easy to find (https://urldefense.com/v3/__https://documentation.sas.com/doc/en/statug/15.2/statug_introcom_sect054.htm__;!!KGKeukY!xhykwhL1jmWXuZyqxtlBEqVv7fDIBEAQmUJ0UbBo8A3o_fvwoLyLuK-OlCLoTVu-l1k4N2Gb9TKpTTak38$ ). I hope the authors find these  resources helpful when experiencing convergence issues in the future. The authors could also have considered using a GEE-type modelhttps://urldefense.com/v3/__https://documentation.sas.com/doc/en/pgmsascdc/9.4_3.3/statug/statug_genmod_examples05.htm__;!!KGKeukY!xhykwhL1jmWXuZyqxtlBEqVv7fDIBEAQmUJ0UbBo8A3o_  fvwoLyLuK-OlCLoTVu-l1k4N2Gb9TK78GI8ww$ ) as the estimates of interest are population-level, and GEEs tend to fit much easier than mixed models.  As I said above, in this particular paper I am satisfied that approaches taken are sufficient and appropriate. However, this would not always be the case, so I wanted to ensure I provided some useful resources for future work.  I also wanted to note that the authors were quite right in their response to item 15, and I thank them for drawing this to my attention. I agree the authors are compliant with the current editorial guidelines, and I have raised my thoughts on this with the editorial team.  Thanks for the opportunity to review and congratulations on an excellent paper. | We thank the reviewer for the comments and for providing references for handling within-individual correlation that is pronounced in future studies. |
| **Reviewer #2** |  |
| Thank you for your thorough responses and the revisions made to the manuscript. I appreciate your efforts to address my concerns, particularly regarding the inclusion of adjusted absolute risks and risk differences, as well as the clarification on the limitations of the data.  While I still have some residual concerns regarding potential confounding by indication and the lack of study-specific data for certain limitations, I recognize that you have made a genuine effort to address these points. I acknowledge the inherent limitations of observational studies and believe  that the remaining aspects are unlikely to significantly alter the overall findings.  Therefore, I strongly recommend that you:  Emphasize the need for caution when interpreting the results in light of the potential confounding by indication, particularly by adding a statement to the conclusion section that acknowledges this potential bias.  I believe that a thorough and transparent discussion of these limitations is crucial for the accurate interpretation of your findings. I recommend acceptance of the manuscript, with these minor revisions. | We thank the review for the comments and have included the suggested language in the Conclusion as follows:  “Caution is needed in interpreting our finding given the limitations of the data and nature of the observational study, which is subject to unmeasured confounding.” (page 21, lines 501-503) |
| **Reviewer #3** |  |
| The authors have appropriately acknowledged the limitations of their work and were responsive to my comments. The remaining thing I struggle with is how physicians who prescribe opioids and antidepressants should interpret this data. At present, discussion of clinical implications is largely absent.  My main question is: Do these results suggest that the opioid + CYP2D6 inhibitor combination is potentially harmful in a clinically meaningful way in older adults in nursing homes? Should clinicians reading this paper consider avoiding such co-prescriptions?  The risks of any medication (i.e., opioids and antidepressants in this case) must be weighed against their potential benefits and alternatives. The effect sizes for many of the risks of opioid and CYP2D6 inhibitor co-prescribing were very small (i.e., RR of 1.04 for pain-related outcomes), and data on potential benefits of co-prescribing was outside the scope of the paper (and thus absent).  If an older adult in a nursing home presents with opioids co-prescribed with CYP2D6 inhibiting antidepressants, I think it would be a stretch to suggest that the data here lend strong support for trying to get patients off the co-prescription.  In conclusion, the data may be statistically significant, but not convincingly clinically significant. | We appreciate the review’s views. In our current version of the conclusion, we do not suggest that clinicians should stop prescribing the CYP2D6-metabolized opioids with the CYP2D6-inhibiting antidepressants. Instead, we suggested that clinicians should increase their awareness among patients who had co-use of these two drugs as follows:  “suggested that clinicians should be aware of potential worsening pain and hospital and ED visits due to pain among patients who concomitantly used CYP2D6-metabolizing opioids and antidepressants, particularly those with CYP2D6-inhibiting antidepressants” ((page 21, lines 498-501)  In this current version, we are also transparent with our study’s limitations by adding the following statement in the Conclusion:  “Caution is needed in interpreting our finding given the limitations of the data and nature of the observational study, which is subject to unmeasured confounding.” (page 21, lines 501-503) |
| **Reviewer #4** |  |
| Thank you for answering my comments and providing additional detailed information on the methods, adjusted risk differences and attenuating the interpretation of the findings increasing the credibility of these kind of studies linking clinical pharmacology with population-based perspectives. | We thank the reviewer for the comments and are happy to know that our response addressed the reviewer’s previous comments. |
| **Editors** |  |
| 1. Please confirm that your abstract complies with our requirements, including providing all the information relevant to this study type   https://urldefense.com/v3/__https://journals.plos.org/plosmedicine/s/submission-guidelines*loc-abstract__;Iw!!KGKeukY!xhykwhL1jmWXuZyqxtlBEqVv7fDIBEAQmUJ0UbBo8A3o_  fvwoLyLuK-OlCLoTVu-l1k4N2Gb9TKupEUR4o$ | We confirmed that our abstract completes the requirements, providing all the information relevant to the study type. |
| 2. Abstract, line 6-7 (minor): add “about”; eg “However, little is known *about* whether and to what extent…” | We have revised the sentence as follows:  “However, little is known about whether and to what extent the…” (page 3, line 6) |
| 3. Please ensure that all abbreviations are defined at first use throughout the text. We prefer that you do not abbreviate “nursing home” throughout the text. | We have ensured that all abbreviations are defined at the first use through the text. We have spelled our ‘nursing home’ in this revision. |
| 4. Please confirm that all numbers presented in the abstract are present and identical to numbers presented in the main manuscript text. | We confirm that all numbers presented in the abstract are present and identical to the numbers presented in the main manuscript text. |
| 5. Please review your text for claims of novelty or primacy (e.g. 'for the first time') and remove or qualify this language. For example, at line 348, you should qualify the statement by inserting the phrase “to our knowledge”. E.g., “This cohort study using a 100% nursing home resident sample linked to their Medicare claims data is, to our knowledge, among the first to provide population-based data…” | Per suggestion, we have revised the sentence as follows”  “This cohort study using a 100% nursing home resident sample linked to their Medicare claims data is, to our knowledge, among the first to provide population-based data”. (Page 17, lines 389-390) |
| 6. In the last sentence of the Abstract Methods and Findings section, please describe the main limitation(s) of the study's methodology. | We have added limitations of the study’s methodology in the Abstract as follows:  “Main study limitations included unmeasured confounding and limited generalizability.” (page 4, lines 35-36) |
| 7. In the author summary, in the final bullet point of 'What Do These Findings Mean?', please include the main limitations of the study in nontechnical language. | Per suggestion, in the author’s summary, we have added the following bullet point:  “The study findings are only generalizable to older nursing home long-term residents and are limited by not fully accounting for confounders, particularly those that cannot be measured in the Medicare and MDS data.” (page 7, lines 81-83) |
| 8. Please state that your study had a prospective statistical analysis plan early in the Methods section, with a call-out to the location of this information in the supplemental information. | We have stated as follows”  “A prospective statistical analysis plan was developed (S1 Text).” (page 9, lines 152-153) |
| 9. In your response to the reviewers, you note that you provide examples of SAS codes for the statistical modelling in your paper in the SAP. Please include some text in the Methods/Statistical analysis section that points readers to the supplement for these examples. | We have added the text in the Methods/Statistical analysis as follows:  “SAS codes for performing outcome regression analyses were provided in S1 Text.” (page 14, line 302) |
| 10. A reminder that all authors must declare their relevant competing interests per the PLOS policy, which can be seen here: https://urldefense.com/v3/__https://journals.plos.org/plosmedicine/s/competing-interests__;!!KGKeukY!xhykwhL1jmWXuZyqxtlBEqVv7fDIBEAQmUJ0UbBo8A3o_   fvwoLyLuK-OlCLoTVu-l1k4N2Gb9TKMvy4-Mc$ For authors with ties to industry,  please indicate whether any of the interests has a financial stake in the results of the current study. | All authors have declared their competing interests per the policy. |
